# Overt Attention toward Appetitive Cues Enhances Their Subjective Value, Independent of Orbitofrontal Cortex Activity

**DOI:** 10.1523/ENEURO.0230-19.2019

**Published:** 2019-10-30

**Authors:** Vincent B. McGinty

**Affiliations:** Center for Molecular and Behavioral Neuroscience, Rutgers University-Newark, Newark, New Jersey 07102

**Keywords:** behavior, electrophysiology, pavlovian conditioning, prefrontal cortex, reward

## Abstract

Neural representations of value underlie many behaviors that are crucial for survival. Previously, we found that value representations in primate orbitofrontal cortex (OFC) are modulated by attention, specifically, by overt shifts of gaze toward or away from reward-associated visual cues (McGinty et al., 2016). Here, we investigate the influence of overt attention on behavior by asking how gaze shifts correlate with reward anticipatory responses and whether activity in OFC mediates this correlation. Macaque monkeys viewed pavlovian conditioned appetitive cues on a visual display, while the fraction of time they spent looking toward or away from the cues was measured using an eye tracker. Also measured during cue presentation were the reward anticipation, indicated by conditioned licking responses (CRs), and single-neuron activity in OFC. In general, gaze allocation predicted subsequent licking responses: the longer the monkeys spent looking at a cue at a given time point in a trial, the more likely they were to produce an anticipatory CR later in that trial, as if the subjective value of the cue were increased. To address neural mechanisms, mediation analysis measured the extent to which the gaze–CR correlation could be statistically explained by the concurrently recorded firing of OFC neurons. The resulting mediation effects were indistinguishable from chance. Therefore, while overt attention may increase the subjective value of reward-associated cues (as revealed by anticipatory behaviors), the underlying mechanism remains unknown, as does the functional significance of gaze-driven modulation of OFC value signals.

## Significance Statement

Recent studies of human decision-making suggest a link between gaze and value: longer fixation of gaze on a given item appears to accentuate its subjective value (its likelihood of being chosen), relative to items that are fixated less. The chief contribution of this study is novel evidence suggesting that gaze also modulates subjective value in simple appetitive conditioning, in an animal model whose gaze behavior closely resembles our own. It is therefore possible that the effects of gaze on value may apply to many forms of motivated behavior. With respect to the neural mechanisms by which gaze influences conditioned responses, our data appear to rule out a role for the OFC, though additional studies are necessary to confirm this finding.

## Introduction

Neural value representations underlie many of the behaviors we rely on to survive, from simple appetitive and defensive reflexes to complex economic decisions. Several recent studies have shown that value representations in the prefrontal cortex can be influenced by how attention is allocated among visual objects of different value. This includes overt shifts of attention (gaze) performed during natural free viewing (McGinty et al., 2016; Hunt et al., 2018) as well as covert shifts of attention performed in the absence of saccadic eye movements (Xie et al., 2018). Allocation of gaze also influences economic choice behavior, with increased gaze time on a given item making it more likely to be chosen over the alternatives (Krajbich et al., 2010; Towal et al., 2013; Vaidya and Fellows, 2015; Gidlöf et al., 2017). A natural hypothesis emerging from these studies is that attention, by modulating neural value signals, may influence a wide range of value-driven behaviors. To test this hypothesis, we build on our recent report of gaze-modulated value signals in the primate orbitofrontal cortex (OFC) during appetitive pavlovian conditioning (McGinty et al., 2016). Whereas the prior report considered only the neural effects of gaze, here we address both the neural and behavioral effects, and ask whether the neural effects are sufficient to explain behavior.

Pavlovian conditioning is a form of learning in which otherwise neutral cues acquire motivational significance (value) after being paired with pleasant or aversive outcomes, so that presentation of the cues alone can elicit conditioned responses (CRs). These responses are usually stereotyped, reflexive behaviors that are performed in direct anticipation of the outcome (e.g., salivation in anticipation of food) and can vary according to the size, probability, frequency, or desirability of the predicted outcome. Our central hypothesis is that overt attention influences CRs performed in anticipation of reward, and that attentional modulation of OFC is the mechanism underlying this influence.

To test this hypothesis, we simultaneously measured pavlovian CRs, eye movements, and OFC neural activity, in an appetitive conditioning task, as described previously (McGinty et al., 2016). We then asked whether trial-by-trial variability in gaze allocation toward the cues corresponded to variability in CR magnitude, and whether this correlation could be statistically explained (mediated) by the firing of single OFC neurons. Although the effect varied according to subject and trial condition, in general, we observed a positive correlation between gaze and CRs: the longer the monkeys spent looking at a pavlovian cue in a given trial, the more likely they were to perform a CR later in that trial, suggesting that gaze allocation influences the in-the-moment subjective value of the cue. With respect to the role of the OFC, we found no evidence that OFC activity could explain the correlation between gaze and CRs, suggesting that some neural substrate outside of the OFC must mediate the influence of gaze on reward anticipation.

## Materials and Methods

### Overview

**Figure 1.**
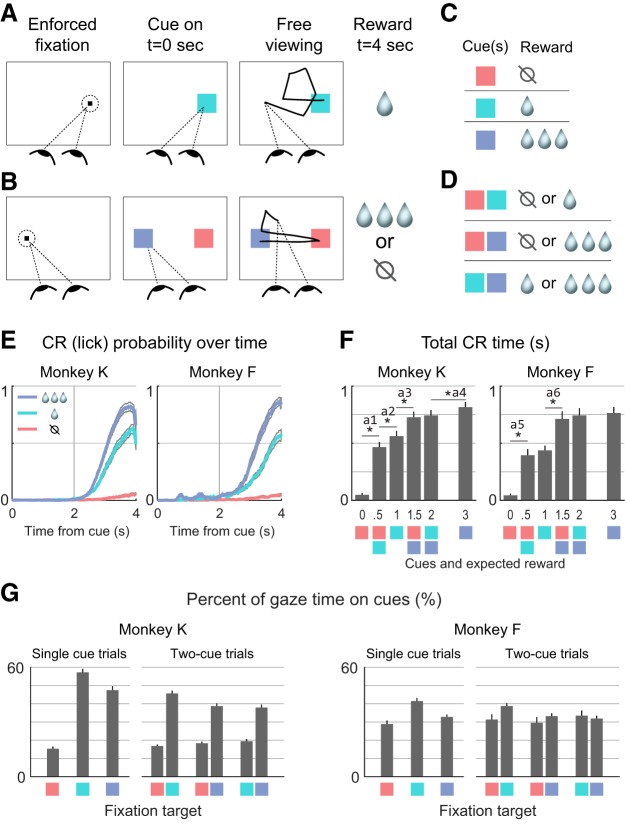
***A–D***, Monkeys were trained to associate visual cues with juice reward: single cues were followed by certain reward (***A***, ***C***); and cues shown in pairs were followed by probabilistic rewards (***B***, ***D***). Anticipatory CRs and allocation of gaze onto the cues were tracked in every trial. ***E***, CRs in single-cue trials. The *y*-axis gives the probability of a CR being detected at a given time point. Colored and gray lines give the mean and 95% confidence intervals, respectively. ***F***, CRs in all trial types. The *y*-axis gives the total time that a CR was detected during cue presentation. The six bars in each graph correspond to the six trial types, with colored squares below the *x*-axis to indicate the cue configuration, and numerals to indicate the mean juice value of the cues. Asterisk indicates significant difference in a comparison between immediately adjacent bars; a1 through a6 refer to entries in Table 1. ***G***, Allocation of gaze onto cues in all trial types. The *y*-axis gives the percentage of time that the gaze was on the cue center (i.e., within 3º). Single-cue trial types are indicated by the three single colored squares, corresponding to the cue value. Two-cue trial types are indicated by adjacent colored squares, corresponding to the cue values shown in that trial type; in these trials, gaze allocation was tallied separately for each cue, hence two bars for each two-cue trial type. Note that gaze allocation was dependent on cue type but was not always a monotonic function of value. Data in ***E–G*** reflect 25 sessions for Monkey K and 28 sessions for Monkey F. Bars show means across sessions, and whiskers show SEM.

**Figure 2.**
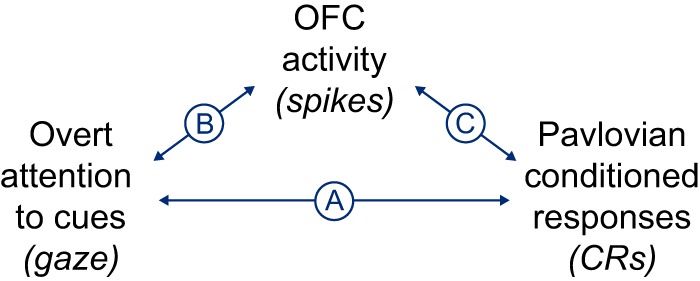
***A–C***, Overview of major analyses: trial-by-trial correlation between gaze allocation and CRs (***A***); modulation of OFC activity by gaze (***B***); and trial-by-trial correlation between OFC activity and CRs (***C***). We also performed a mediation analysis, which quantifies the degree to which the gaze–CR correlation (***A***) can be statistically explained by the combined effect of gaze on OFC activity and the correlation between OFC activity and CRs (***B***, ***C***).

Macaque monkeys performed an appetitive pavlovian conditioning task (Fig. 1*A*), while the following three variables were measured simultaneously: allocation of gaze (overt attention), reward anticipation, and value representations in OFC (Fig. 2). Gaze was measured relative to the location of reward-predictive pavlovian cues, and gaze allocation was quantified as the fraction of time the monkeys spent looking at the cues. Reward anticipation was defined as the conditioned licking responses (CRs) that monkeys performed in the moments leading up to reward delivery, and was quantified as the fraction of time that a CR response was detected. OFC value representations were measured on the basis of single and multiunit neural activity (see Analysis below).

**Figure 3.**
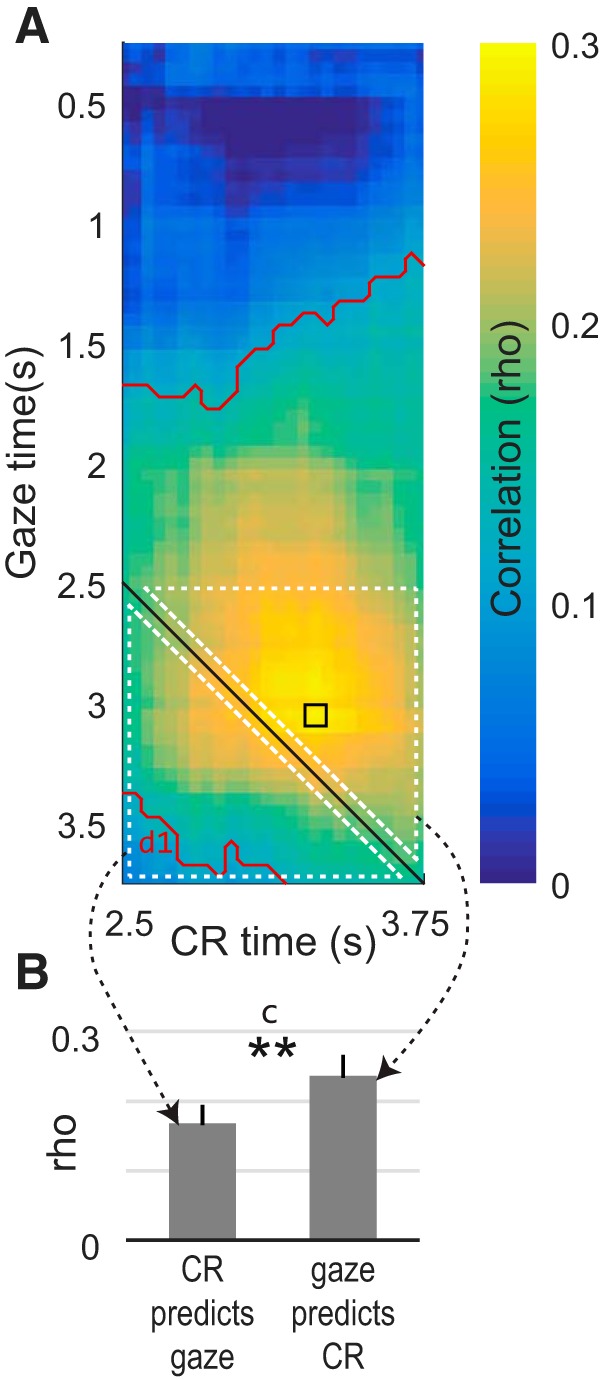
Gaze allocation predicts CRs in example data from a single trial type in one monkey. ***A***, The heatmap shows the trial-by-trial correlation between the time spent looking at a cue and the time spent performing an anticipatory CR, assessed across different time points in the trial. Warmer colors indicate that greater gaze allocation was associated with more CRs. The *x*- and *y*-axes give the times at which the CR and attentional data were observed, respectively, and equivalent time points are given by the black diagonal. The small black square shows the peak correlation. Red contours indicate mean values significantly above zero; d1 refers to corresponding entry in Table 2. White dotted triangles indicate time points averaged together to produce the bar graphs in ***B***. ***B***, Bar heights give the average correlation within the white-outlined pixels above and below the black diagonal in ***A***. Whiskers give the SEM; **indicates significant difference between the bars; c refers to the corresponding entry in Table 1.

**Figure 4.**
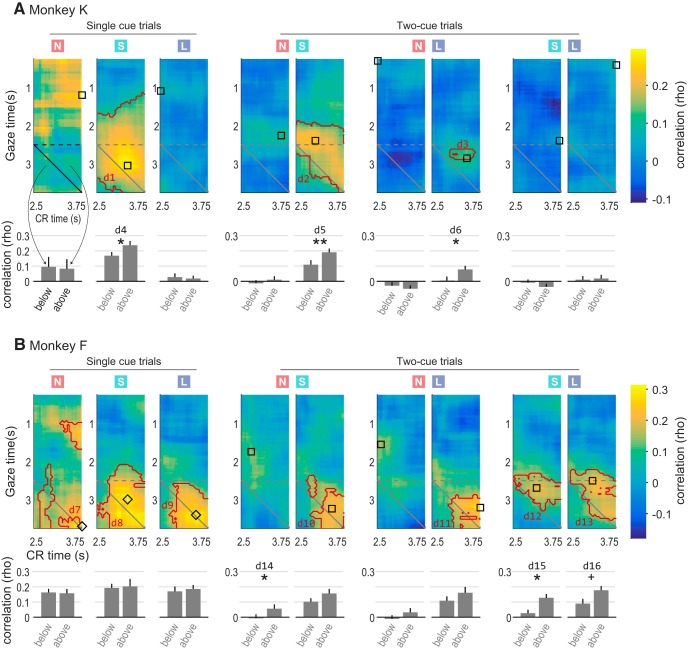
Gaze–CR correlation in all conditions for Monkey K (***A***) and Monkey F (***B***). The colored squares at top indicate whether the gaze data pertain to “none”, “small”, or “large” cues (red N, green S, and blue L, respectively). Note that in two-cue trial types, gaze is assessed separately for each cue; hence, there are two conditions for each two-cue trial type. Heatmap colors and contours follow the same conventions as in Figure 3*A*, and bar graphs below each heatmap follow the same conventions as in Figure 3*B*. The black square or diamond on each map indicates the time point used in subsequent analyses of how OFC activity correlates with CRs (see main text and Fig. 8). Squares show the point of highest gaze–CR correlation in the map, and diamonds show the highest point above the main diagonal (see Materials and Methods). Symbols * and ** indicate significance between adjacent bars; + indicates marginally significant difference; d1 through d16 refer to the corresponding entries in Table 1 and Table 2. The data from Figure 3 are reproduced in the first row, second column (single “small” cue trials in Monkey K).

The analyses had two main objectives. The first was to determine whether gaze allocation and reward anticipation were correlated with one another on a trial-by-trial basis. The second was to determine whether this correlation could be explained, in a statistical sense, by the activity of OFC neurons. To satisfy the first objective, we computed the correlation between the time spent looking at pavlovian cues and the duration of CRs across trials (Fig. 2*A*). Importantly, the relatively long trial duration (4 s) allowed the correlation to be assessed across different time points in the trial; thus, we were able to determine whether looking at a cue early in the trial was correlated with CRs later in the trial (i.e., whether gaze allocation could predict subsequent reward anticipation). The results are shown in Figures 3 and 4.

**Figure 5.**
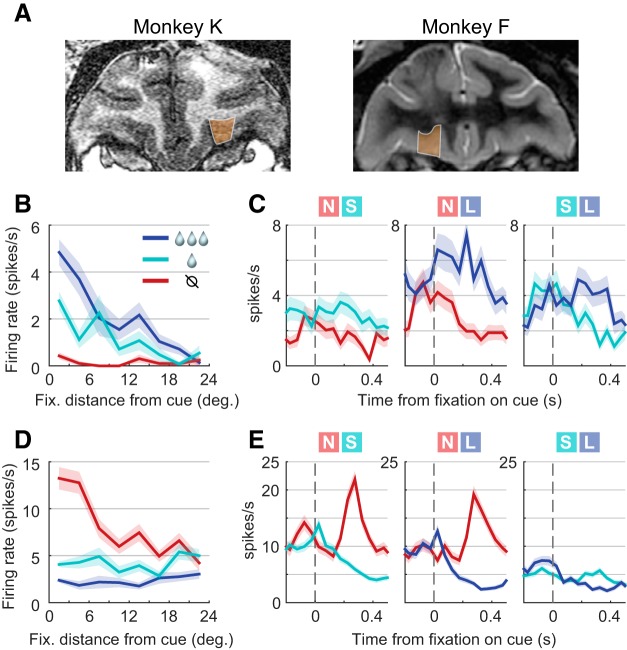
Value signals in OFC neurons were modulated by shifts of gaze toward or away from the cues. ***A***, Coronal MRI sections from the two subjects; orange shading shows the recorded areas. ***B***, Single-example cell. In single-cue trials, firing increased as a function of cue value (colors) and decreased as a function distance of fixation from the cue (*x*-axis). In addition, the effect of value was maximal for fixations on or near the cue, constituting an interaction between the value and distance effects. ***C***, Same cell as in ***B***. During two-cue trials, firing was greater following fixations onto the higher of the two cue values shown. Each of the three panels shows firing time locked to fixation onset at *t* = 0; the trial type (cues shown) is at top, and the line color indicates which of the two cues was fixated. Lines give the means, and shading gives the SEM. ***D***, ***E***, A second example cell, showing effects of value and attention (including interaction effects) on firing in single-cue trials (***D***) and two-cue trials (***E***). Unlike the cell in ***B*** and ***C***, firing decreases as a function of the single-cue value or of the fixated cue value in two-cue trials. deg., Degree; Fix., fixation.

**Figure 6.**
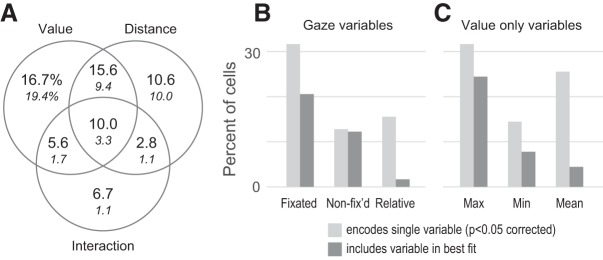
Modulation of OFC firing by gaze. ***A***, OFC activity in single-cue trials was explained by a linear model with three regressors: cue value, distance of fixation from the cue, and their interaction. The fraction of cells with significant effects are shown in the Venn diagram, using uncorrected and corrected thresholds of *p* < 0.05 (large and small numerals, respectively). ***B***, ***C***, OFC activity in two-cue trials was explained using a series of single- and two-variable models fit to every cell (Table 3). The light gray bars give the fraction of cells that encode each variable when fit by itself (of *n* = 180 cells, *p* < 0.05 corrected). The dark gray bars give the fraction of cells that include a given variable in its best fitting model. Fix’d, Fixated; Max, maximum; Min, minimum.

**Figure 7.**
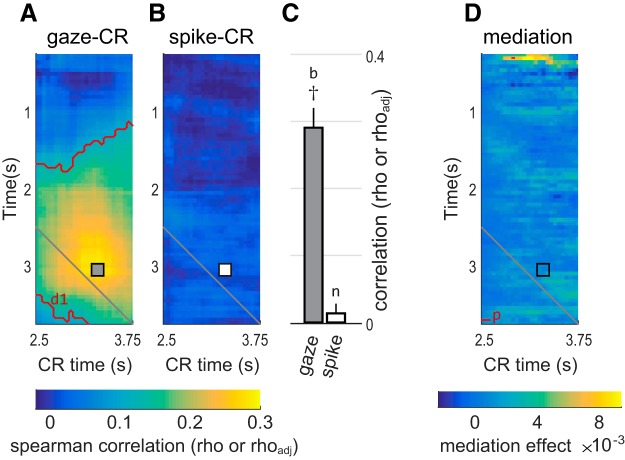
OFC activity does not predict CRs and does not mediate the effects of gaze on CR, in example data from single “small” cue trials in Monkey K. ***A***, The average gaze–CR correlation (rho) in single “small” cue trials in Monkey K, reproduced from Figure 3, using the same conventions. The gray square shows the pixel with the highest correlation. This same pixel is marked with a square in ***B*** and ***D***. Red contours indicate significant correlations, as in Figure 3; d1 refers to corresponding entry in Table 2. ***B***, The cell-averaged spike–CR correlation (rho_adj_), using data from the same trial type and monkey as in ***A***. The white square corresponds to the peak gaze–CR effect in ***A***. ***C***, The left and right bars give the mean correlations at the pixels marked with the square in ***A*** and ***B***, respectively. Whiskers indicate SEM. Dagger (†) indicates that the point lies within the red significance contours in ***A***. b and n refer to corresponding entries in Table 1. These data are reproduced in Figure 8*A*, alongside data from all conditions in both monkeys. ***D***, Average mediation effects for single “small” cues in Monkey K. See main text for details. Red contours indicate median mediation effects above zero at *p* < 0.001 (uncorrected); p refers to the corresponding entry in Table 2. Mediation effects take the same units as the regression estimates (β values) on which the analysis is based (see Materials and Methods).

**Figure 8.**
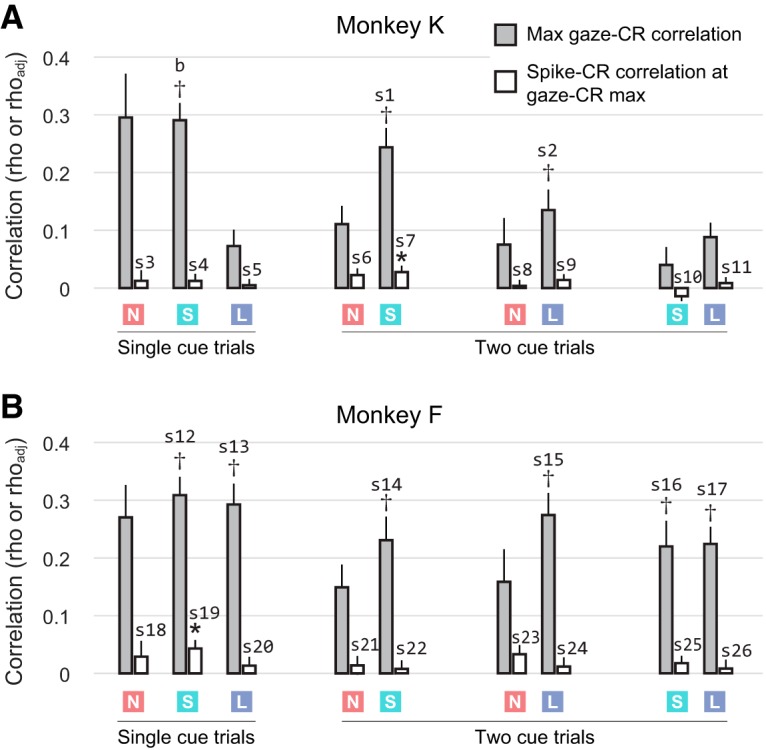
OFC neural activity is not correlated with CRs. Each pair of bars corresponds to one heatmap in Figure 4. The left bar gives the gaze–CR correlation at the time bins for which this effect was maximal (Fig. 4, black squares); and the right bar gives the spike–CR correlation at these same time bins. The colored squares indicate whether the data pertain to “none”, “small”, or “large” cue values (red N, green S, and blue L, respectively). For two-cue trial types, the effects are assessed separately for each cue; hence, there are two sets of results for each two-cue trial type. Panel ***A*** shows data from Monkey K, and panel ***B*** shows data from Monkey F. The data from Figure 7*C* are reproduced in ***A*** (single “small” cue trials). Whiskers give SEM. Daggers (†) indicate that the peak gaze–CR effect falls within the significance contours in Figure 4; details for these within-contour peak effects are provided in Table 1 in b, s1–s2, and s12–s17. For the spike–CR data, * indicates a significant difference from zero. Details are provided for all spike–CR effects in this figure to show negative results; see Table 1 s3–s11 and s18–s26.

To satisfy the second objective required testing the following two correlational relationships: between gaze and OFC activity (Fig. 2*B*), and between OFC activity and CRs (Fig. 2*C*). The relationship between gaze and OFC activity was assessed using linear models, as in our prior work (McGinty et al., 2016; Figs. 5, 6, results). To quantify the relationship between OFC firing and CRs, we used a modified form of Spearman’s correlation coefficient (Figs. 7, 8, results). Finally, to quantify the degree to which OFC firing could statistically explain the gaze–CR correlation, we performed a mediation analysis (results in main text).

Most analyses were performed separately for each trial type; for example, trials with a single “none” cue were analyzed as a group, separately from trials with a single “small” reward cue, and separately from trials with both a “none” and “small” reward cue shown simultaneously. This was done because the correlation between gaze and CRs (the key behavioral outcome in this study) differed between trial types, as we illustrate below. However, to identify OFC cells modulated by gaze, all single-cue trial types were analyzed together, and all two-cue trial types were analyzed together. This was done to assess the encoding of value, which could only be done by comparing firing across trials with differing cue value (i.e., different trial types).

Key statistical results are described in Table 1 and Table 2. The rows of the tables are named with lower case letters (e.g., a, b, c), which correspond to superscript indicators in the main text and to text indicators embedded in the figures. The *p* value corrections for multiple tests are performed within subject, using Holm’s variant of the Bonferroni correction; any corrected *p* values >1 are set to exactly 1.

**Table 1:** Statistical table

Identifier	Test used	Number of observations	*p* Value
a1	Paired signed rank test	25 sessions	1.2 * 10^−5^
a2	Paired signed rank test	25 sessions	0.0027
a3	Paired signed rank test	25 sessions	8.9 * 10^−4^
a4	Paired signed rank test	25 sessions	1.0 * 10^−4^
a5	Paired signed rank test	28 sessions	3.8 * 10^−6^
a6	Paired signed rank test	28 sessions	7.3 * 10^−6^
b	One-sample *t* test	25 sessions	3.2 * 10^−10^
c	Paired *t* test	25 sessions	0.002
d4	Paired *t* test with Holm–Bonferroni correction, 9 comparisons	25 sessions	0.017
d5	Paired *t* test with Holm–Bonferroni correction, 9 comparisons	23 sessions	0.0068
d6	Paired *t* test with Holm–Bonferroni correction, 9 comparisons	25 sessions	0.017
d14	Paired *t* test with Holm–Bonferroni correction, 9 comparisons	23 sessions	0.032
d15	Paired *t* test with Holm–Bonferroni correction, 9 comparisons	27 sessions	0.032
d16	Paired *t* test with Holm–Bonferroni correction, 9 comparisons	27 sessions	0.058
e	One-sample *t* test	180 cells	4.3 * 10^−6^
f	One-sample *t* test	180 cells	0.53
g	Χ^2^ test for association	180 cells	1.2 * 10^−7^
h	Χ^2^ test for association	180 cells	1.4 * 10^−5^
i	Pearson's correlation	180 cells	1.6 * 10^−15^
j	Pearson's correlation	180 cells	3.6 * 10^−30^
k	Pearson's correlation	180 cells	1.6 * 10^−26^
n	One-sample *t* test	111 cells	0.26
q	One-sample signed rank test	114 cells	0.3
r	One-sample signed rank test	54 cells	0.043
s1	One-sample *t* test	23 sessions	1.5 * 10^−7^
s2	One-sample *t* test	25 sessions	5.8 * 10^−4^
s3	Paired *t* test with Holm–Bonferroni correction, 9 comparisons	19 cells	1
s4	Paired *t* test with Holm–Bonferroni correction, 9 comparisons	111 cells	1
s5	Paired *t* test with Holm–Bonferroni correction, 9 comparisons	114 cells	1
s6	Paired *t* test with Holm–Bonferroni correction, 9 comparisons	102 cells	0.26
s7	Paired *t* test with Holm–Bonferroni correction, 9 comparisons	102 cells	0.049
s8	Paired *t* test with Holm–Bonferroni correction, 9 comparisons	105 cells	1
s9	Paired *t* test with Holm–Bonferroni correction, 9 comparisons	114 cells	0.67
s10	Paired *t* test with Holm–Bonferroni correction, 9 comparisons	113 cells	0.35
s11	Paired *t* test with Holm–Bonferroni correction, 9 comparisons	112 cells	1
s12	One-sample *t* test	24 sessions	5.5 * 10^−10^
s13	One-sample *t* test	26 sessions	8.7 * 10^−9^
s14	One-sample *t* test	23 sessions	6.7 * 10^−6^
s15	One-sample *t* test	26 sessions	8.9 * 10^−8^
s16	One-sample *t* test	26 sessions	3.0 * 10^−5^
s17	One-sample *t* test	26 sessions	2.9 * 10^−8^
s18	Paired *t* test with Holm–Bonferroni correction, 9 comparisons	24 cells	1
s19	Paired *t* test with Holm–Bonferroni correction, 9 comparisons	55 cells	0.021
s20	Paired *t* test with Holm–Bonferroni correction, 9 comparisons	59 cells	1
s21	Paired *t* test with Holm–Bonferroni correction, 9 comparisons	52 cells	1
s22	Paired *t* test with Holm–Bonferroni correction, 9 comparisons	53 cells	1
s23	Paired *t* test with Holm–Bonferroni correction, 9 comparisons	57 cells	0.23
s24	Paired *t* test with Holm–Bonferroni correction, 9 comparisons	56 cells	1
s25	Paired *t* test with Holm–Bonferroni correction, 9 comparisons	59 cells	0.9
s26	Paired *t* test with Holm–Bonferroni correction, 9 comparisons	59 cells	1
t	Pearson’s correlation	180 cells	2.4 * 10^−4^
u	Pearson’s correlation	180 cells	5.9 * 10^−4^
v	Pearson’s correlation	180 cells	7.5 * 10^−5^

Identifiers refer to superscript identifiers in main text or to text identifiers placed within figures. For some tests, the number of sessions or cells used may be less than the total number of sessions or cells in the study. This can occur because data in some pixels in some sessions are removed (set to nan), as a quality control measure (see Materials and Methods).

**Table 2:** Statistical table for significance contours in Figures 3, 4, and 7

Identifier	Minimum number of observations	Maximum number of observations	Largest cluster size (pixels)
d1	25 sessions	25 sessions	1137
d2	23 sessions	23 sessions	643
d3	25 sessions	25 sessions	55
d7	13 sessions	13 sessions	123
d8	22 sessions	25 sessions	612
d9	25 sessions	26 sessions	378
d10	23 sessions	23 sessions	214
d11	26 sessions	26 sessions	217
d12	25 sessions	27 sessions	250
d13	26 sessions	27 sessions	363
p	70 cells	114 cells	3

With the exception of entry p (corresponding to Fig. 7*D*), all contours are calculated on the basis of one-sample *t* tests at an initial threshold of *p* < 0.001, with cluster correction at a FWER of *p* < 0.01. Within a significance contour, the number of sessions or cells used to calculate the mean effect at any given pixel may be less than the total number of sessions or cells in the study. This can occur because data in some pixels in some sessions are removed (set to nan), as a quality control measure (see Materials and Methods). For each contour region, the columns for the Maximum and Minimum number of observations therefore give the number of observations for the pixel with the smallest and largest number used, respectively

### Subjects and apparatus

All procedures were performed in accordance with the National Institutes of Health *Guide for the Care and Use of Laboratory Animals* and were approved by the Animal Care and Use Committee of Stanford University, where the data were collected. The subjects were two adult male rhesus monkeys designated K and F, weighing 13.5–15.0 kg. They were implanted with an MR-compatible head holder, and subsequently with a recording chamber (Crist Instruments); a craniotomy was also performed to allow access to the OFC. All surgical procedures were performed under full surgical anesthesia using aseptic techniques and instruments, with analgesics and antibiotics given preoperatively, intraoperatively, and/or postoperatively, as appropriate. Data were collected while the monkeys were head restrained and seated ∼57 cm from a frontoparallel CRT monitor displaying the task stimuli. The stimuli were square color patches (3.2º per side) and were mutually isoluminant. Horizontal and vertical eye positions were recorded at 400 Hz. A tube for fluid rewards was placed outside the mouth, and to retrieve an available reward the monkeys had to touch their tongue to the end of the tube during delivery. Both monkeys quickly learned to do so and typically consumed all of the juice delivered on every trial. Monkeys typically performed anticipatory conditioned licking responses before reward delivery, and these were quantified according to the fraction of time that a response was detected in a given epoch (see below).

Task flow and stimulus presentation were controlled using the REX software suite (Laboratory of Sensorimotor Research, National Eye Institute) and dedicated graphics display hardware (Cambridge Research Systems). Neural signals were measured from single tungsten electrodes (FHC) placed at the target locations using a motorized drive (NAN Instruments). Neural activity, eye position, and task event data were acquired and stored using the MAP Data Acquisition System (Plexon).

### Behavioral task

The task was identical to that used in McGinty et al. (2016), with the exception that on some trials, two cues were shown simultaneously. See Figure 1*A–D* for an illustration, and below for details. The monkeys were trained to associate three different color cues with three juice rewards in approximate ratios of 3:1:0. These are referred to as “large,” “small,” and “none,” or as “L,” “S,” and “N,” and they are indicated in the figures by the colors blue, turquoise, and red. Juice volumes were constant within a session, but varied slightly across sessions to compensate for changes in the fluid sensitivity of the monkeys during the study. A session was defined as the behavioral and neural data collected on a single day; more than one cell was typically recorded in each session. Only sessions with concurrently recorded neural data were used.

Trials began with a fixation point (FP) appearing 5º to the left or right of the screen center. After the monkey fixated on this point for 1–1.5 s, either one or two cues were shown, at which point the monkey was free to move his eyes. Eye position was monitored but had no consequence for trial outcome. Reward was delivered 4 s after cue onset, depending on which cue or cues were shown (see below). The cues were extinguished at 4.3 s after cue onset, after which there was a 2–4 s intertrial interval, followed by the illumination of the FP on the next trial.

Trials had either a single cue or two different cues shown simultaneously. In single-cue trials (Fig. 1*A*), one randomly chosen cue appeared at the location of the FP, and the volume of reward delivered at the end was determined by the color of the cue (Fig. 1*C*). In two-cue trials, one randomly selected cue appeared at the FP location (5º left or right of center), and a different randomly selected cue appeared 5º from center in the opposite direction of the FP location (Fig. 1*B*). At the end of the trial, one of the two reward volumes was randomly chosen to be delivered (Fig. 1*D*). For example, the trial illustrated in Figure 1*B* has a “large” and “none” cue, indicating a 50% probability of a large reward and a 50% probability of no reward. Single-cue and two-cue trials types were presented in equal proportions, randomly interleaved within a session.

New cue colors were selected for every session by randomly sampling equidistant points on a color wheel. Each session therefore began with a learning phase, which was completed before data collection. During learning, single-cue trials were presented until the CR performed by monkey during cue presentation (the 4 s before reward) became proportional to the reward size: “large” > “small” > “none” with the CR for “none” trials being absent or negligible. Learning was considered to be complete when the CR durations were significantly different (rank sum test, *p* < 0.01 uncorrected; analyzed on-line and not shown here) over the previous 60–100 learning trials. Learning phase data were not used in any analysis in this article. In a prior report from this dataset (McGinty et al., 2016), we examined the effects of cue–reward “reversals” on some OFC cells. Those cells are also used here; however, for a given cell we use only the pre-reversal or post-reversal data (never both) according to whichever segment of the data had more trials. In other words, in this report the cue–reward associations were static, with no reversals. In Monkey F, all cells used prereversal data only (*n* = 64), and in Monkey K, 69 of 116 cells used prereversal data and the remaining 47 used postreversal data. The results for Monkey K did not differ between the cells using prereversal and postreversal data (data not shown).

### Conditioned responses and quantification of reward anticipation

Monkeys typically performed CRs during the 4 s cue display period in anticipation of reward delivery. CRs were quantified by detecting the presence/absence of contact between the tongue and juice delivery tube. This was done by connecting the input lead of a single-channel amplifier (400 Hz sampling; A-M Systems) to the fluid reservoir, and the ground lead to the seat of the task chair. Tongue contact with the juice tube abruptly reduced the amplitude of ambient noise on the channel, and setting an appropriate noise threshold effectively binarized the signal into epochs of contact/no contact. The CR versus time plots in Figure 1*E* show the proportion of trials in which contact was present at a given time point. Total contact time throughout the trial was averaged across trials of a particular type to produce Figure 1*F*. For the main analyses in this study, CR data were first segmented into overlapping bins, each 500 ms in duration, with 50 ms increments between bin centers. The first bin was centered at 2500 ms after cue onset, because CRs were nearly always absent until that time (Fig. 1*E*). The last bin was centered at 3750 ms after cue onset, for a total of 26 bins. CRs were then quantified by finding the fraction of time within each 500 ms bin that contact was detected.

### Eye tracking and quantification of gaze

Gaze was unrestricted during the 4 s cue display period. Horizontal and vertical eye positions were recorded at 400 Hz using a noninvasive optical system in Monkey K (EyeLink, SR Research) and a scleral search coil system in Monkey F (C-N-C Engineering). These different eye-tracking methods yield similar data (Kimmel et al., 2012).

Gaze location was quantified in relation to the cue or cues. For analyses of gaze effects on CRs and on neural activity, gaze data were segmented into overlapping bins (500 ms each, 50 ms increments between bin centers), over the 4 s cue presentation period, yielding a total of 71 bins, with the first centered at 250 ms after cue onset and the last centered at 3750 ms. Gaze allocation for each bin was quantified as the fraction of time (of 500 ms) that gaze was within 3º of the center of a cue (“on” the cue). In two-cue trial types, gaze allocation was tallied for each cue individually.

For the analysis of gaze effects on spiking, OFC neural activity was analyzed with respect to gaze location. Here, it was necessary to time lock neural data to gaze behavior to create a temporal reference point for perievent time histograms, and to account for the known temporal lag between visual events and OFC activity (Thorpe et al., 1983; Wallis and Miller, 2003; Padoa-Schioppa and Assad, 2006). Therefore, eye position data were segmented into fixation and saccade epochs (Engbert and Kliegl, 2003; Kimmel et al., 2012), and the fixation onsets were used as the temporal reference point for spiking data, as in our prior work (McGinty et al., 2016). To assess neural data with respect to fixation location in single-cue trials, fixations were quantified according to the distance of gaze from the cue center, which is consistent with our prior report. In two-cue trial types, fixations away from the cues were infrequent, and so neural analyses only used data from “on-cue” fixations (within 3º of the cue center).

### Neural recordings

Single electrodes were introduced into the brain through a sharpened guide tube whose tip was inserted 1–3 mm below the dura. OFC was identified on the basis of gray/white matter transitions, and by consulting a high-resolution MRI acquired from each animal after chamber implantation. We targeted the fundus and lateral bank of the medial orbital sulcus and the laterally adjacent gyrus, corresponding approximately to Walker’s area 13 (Ongür and Price, 2000).

From Monkey K, we recorded 116 neural unit signals (“cells”) over 25 sessions; and from Monkey F, we recorded 64 cells over 28 sessions. (Only sessions with concurrently collected neural data were used.) These included putative single units, characterized by large and well isolated waveforms (*n* = 63 from Monkey K; *n* = 44 from Monkey F), as well as multiunit signals with low amplitude, poorly isolated waveforms (53 from Monkey K; 20 from Monkey F). Among the single units, some were isolated during the learning phase of the task (see above) and were selected for subsequent recording because they showed an apparent increase or decrease in firing during cue presentation; the remainder of the single unit signals, and all multiunit signals, were recorded without any prior observation of their activity during task performance. Neural data were only recorded and analyzed after the learning phase was complete. In some sessions, several cells were recorded simultaneously by isolating more than one cell on a given electrode and/or by using two electrodes at once. See the study by McGinty et al. (2016) for details.

After data collection, spikes were assigned off-line to individual units based on the principal component features of the waveforms (Offline Sorter 2.0, Plexon). On rare occasions, cells initially designated as single units were recategorized as multiunit if they showed an abundance of short interspike intervals (>0.05% of intervals <2 ms). After unit sorting, the data were imported into MATLAB and the R software environment for analysis. There were no major differences in results obtain from single and multiunit signals, and so their data are presented together.

### Analysis

#### Correlation between gaze allocation and reward anticipation

The objective of this analysis was to assess the trial-by-trial correlation between the fraction of gaze time devoted to pavlovian cues and the fraction of time spent performing CRs in anticipation of reward delivery. Gaze and CR data were calculated in 500 ms bins (50 ms increments). Within each session, the across-trial correlation was calculated for all possible pairs of bins, and these correlations were then averaged across the sessions for each monkey (25 for Monkey K; 28 for Monkey F). The resulting matrix of correlations has rows and columns corresponding to the bin centers for gaze and CR data (respectively).

The correlation statistic was Spearman’s rho, a nonparametric, outlier-resistant, ranks-based measure of association. It is preferred over Pearson’s *r* because the data were not normally distributed: measurements of gaze and CR durations were bounded by the 500 ms bin size and had a Bernoulli-like distribution; and the spiking data (below) naturally took on a Poisson-like distribution. As a quality control measure, no correlation was calculated (i.e., the correlation was set to “nan”) when >80% of the gaze data or >80% of the CR data within a given bin had the same value; this happened most frequently in single “none” value trials, where the CR was often absent.

Correlation matrices are displayed as heatmaps showing the average correlation across sessions. One heatmap was calculated per single-cue trial type, and for each two-cue trial type one heatmap was calculated for the lower value cue and another for the higher value cue.

At each point on the map, the average correlation was compared with zero by means of a *t* test. Red contours show points that surpass both an initial significance threshold of *p* < 0.001 as well as a cluster-extent threshold of *p* < 0.01 to control for multiple comparisons. The cluster extent threshold was determined as follows: for every map, we created 1000 “null” correlation maps using data in which the trial labels for the gaze data were randomly shuffled within each trial type. The null maps were thresholded at *p* < 0.001, and the largest group of contiguous significant pixels (maximum cluster size) was recorded for each null map. (Contiguity was defined as a shared edge; shared corners were not considered contiguous.) This produced a distribution of 1000 maximum cluster sizes under the null hypothesis (no gaze–CR correlation). The cluster extent threshold was set to the 10th largest maximum cluster size (top 1.0 percentile). Then, in the original data, all clusters of significant points smaller than this threshold were discarded, corresponding to a cluster-level familywise error rate (FWER) of *p* < 0.01.

#### Gaze modulation of OFC neural activity

The objective of this analysis was to quantify the fraction of OFC neurons that are modulated by shifts of gaze toward or away from the pavlovian cues. Single-cue trials were analyzed as a group, separately from two-cue trials.

##### Data source

The 180 neurons analyzed here are the subset of the 283 neurons analyzed in the study by McGinty et al. (2016) for which both the single-cue and two-cue data were collected. For single-cue trials, the main analyses in the study by McGinty et al. (2016) are repeated here, and so the data reported (Figs. 5*B*,*D*, 6*A*) are in essence a restatement of the earlier findings, in a subset of the original data. The two-cue trial data were obtained from these same 180 neurons, but have not been published before, with the exception of preliminary analyses in abstract form (McGinty et al., 2014).

##### Single-cue trials

The basic unit of data was fixation-evoked firing, which was defined as the spike count observed 100–300 ms after the beginning of each fixation epoch (see above). The temporal offset accounts for the typical delay in OFC responses to visual stimuli (Thorpe et al., 1983; Wallis and Miller, 2003). This time window captures the peak fixation-evoked response in gaze-responsive OFC cells, as illustrated in the study by McGinty et al. (2016, their Fig. S4C).

For every neuron, we fit the GLM in Equation 1. Cells with significant effects were identified for each regressor (*p* < 0.05 both uncorrected and corrected with Holm’s Bonferroni correction). The GLM assumed a negative binomial error model, and is given by the following:

(1) $$\text{log}\;\left(\text{Y}\right)=\beta_{0}+\beta_{\text{VAL}}*\text{Value}+\beta_{\text{DIST}}*\text{Distance}+\beta_{\text{VALxDIST}}*\text{Val}\times \text{Distance},$$

where each observation is a fixation (as defined above), *Y* is the gaze-evoked spike count for that fixation, Value refers to the volume of juice associated with the cue in each trial, Distance refers to the distance of gaze from the cue center for each fixation, and Val × Distance is the interaction of the Value and Distance variables (computed after centering them).

##### Two-cue trials

We focused on firing evoked by on-cue fixations (<3º from cue center), due to the low frequency of off-cue fixations (Fig. 1*G*). To assess gaze and value effects, for every cell we fit GLMs that explained fixation-evoked firing on the basis of six variables (Table 3, columns). Three variables describe a value signal modulated by shifts of gaze between cues (i.e., a pattern of firing that depends not only on the values of the cues shown, but also on which cue is fixated at any given moment). They are as follows: (1) the value of the fixated target; (2) the value of the other (nonfixated) target shown; and (3) the relative value of the fixated target, defined as the fixated minus nonfixated target value, suggested by recent findings in frontal lobe recordings (Hunt et al., 2018). The three other variables describe a value signal with no modulation by gaze, as follows: (4) the maximum of the two cue values shown; (5) the minimum of the two shown; and (6) the mean of the two shown.

**Table 3:** Models used to explain firing in two-cue trials

	Gaze-dependent variables			Non-gaze-dependent variables			
Model	Fixated target	Nonfixed target	Relative value	Maximum value	Minimum value	Mean value	Number of cells best fit by each model
1	x						5
2		x					2
3			x				3
4				x			8
5					x		0
6						x	8
7	x	x					6
8	x			x			20
9	x				x		6
10		x		x			11
11		x			x		3
12				x	x		5

Each row specifies a model, which can use either one or two variables. The variables included in a given model are indicated by “x.”

Because these six variables are not linearly independent, they cannot be assessed simultaneously in the same model. For example, the “mean value” variable (6 above) is a linear combination of the “maximum value” and “minimum value” variables (4 and 5 above), meaning that independent estimates for these three variables cannot be obtained from a single model. We therefore adopted a competitive modeling approach, in which we fit a set of models containing either one or two regressors (see Table 3, rows 1–12) and then identified the best fitting model for each cell using the Akaike information criterion (AIC). The results were quantified by finding the percentage of cells with significant effects of each single variable when fit by itself (Fig. 6*B*,*C*, light bars), as well as the percentage of cells that included a given variable in its best-fit model (Fig. 6*B*,*C*, dark bars).

The set of tested models is shown in Table 3. Other variable combinations were not tested due to the linear dependence of the variables. In brief: all models with more than four variables and some with three variables were excluded due to the strict linear dependence of the regressors, as in the example above. The subset of three-variable models that were able to be fit could not be distinguished from one another in terms of goodness of fit, because they all explained the same portion of variance in the data (again due to the nonindependence of the regressors) and, therefore, yielded the same AIC. (In linear algebra terms, the matrix of regressors for fitable three-variable models all shared the same basis.) Some two-variable models also yielded nonunique AICs for the same reason and were also excluded. Thus, Table 3 shows all the combinations of the six regressors that can be fit in a single model and that also uniquely explain variance in firing, and can therefore be compared in goodness-of-fit terms.

#### Correlation between OFC activity and reward anticipation

The objective of this analysis was to assess the trial-by-trial correlation between the activity of OFC neurons and the fraction of time spent performing CRs in anticipation of reward delivery. This procedure is similar to the gaze–CR correlation calculation described above. Spiking and CR data were calculated in 500 ms bins (50 ms increments), and the correlation was performed across trials for all possible pairs of bins, yielding a matrix of correlation coefficients. No correlation was calculated (dataset to nan) when >80% of the spike data or >80% of the CR data in a given bin had the same value. Correlations were calculated individually for each OFC cell (*n* = 116 for Monkey K; *n* = 64 for Monkey F), and then averaged across cells. Six correlation matrices were calculated for each cell, one for each of the six trial types shown in Figure 1, *C* and *D*.

Unlike the gaze–CR calculation, the correlation statistic was an unsigned variable that we term the “absolute adjusted correlation”, defined as follows:

(2) $$rho_{\mathrm{adj}}=\mathrm{abs}\left(\mathrm{rho}\right)-<rho_{\mathrm{null}}>,$$

where abs(rho) is the absolute value function, and rho is the raw Spearman’s correlation between the spike count and the CR. To find <rho_null_>, we randomly shuffle the trial labels 100 times within a cell, find the absolute Spearman’s correlation in each shuffle, and take the mean across shuffles. Thus, <rho_null_> is the absolute correlation that would be expected under the null hypothesis that spiking and the CR are unrelated; it is always above zero, because even totally random data produce spurious nonzero correlations. The rationale for using rho_adj_ is as follows: first, taking the absolute value of the raw spike–CR correlation puts positive spike–CR relationships (more spiking/more CR) on the same scale as cells that have a negative spike–CR relationship (more spiking/less CR); this allows us to average the correlations across all cells, regardless of the sign of the effect. Second, by subtracting <rho_null_>, the value of rho_adj_ is expected to be zero for cells in which there is no relationship between spiking and the CR, but is expected to be positive for cells in which there is a spike–CR relationship. The across-cell mean of rho_adj_ can be therefore assessed by a *t* test versus zero, to determine whether a reliable spike–CR relationship exists at the population level.

The heatmap in Figure 7*B* shows the rho_adj_ for one trial type in one monkey, calculated at all pairs of time bins, averaged across all cells. The heatmap was thresholded at *p* < 0.001, but there were no significant pixels that survived cluster correction to an FWER of *p* < 0.01.

In the bar graphs in Figure 8, the cell-averaged rho_adj_ value is shown for all trial types and both monkeys, but at only a single point on the heatmap, which was selected as follows: for each of the gaze–CR heatmaps in Figure 4, we selected the point with the highest average correlation (black squares). In almost all trial types, this peak point was above the diagonal, reflecting the fact that overt attention (gaze) tends to predict subsequent CRs. However, in the three single-cue trial types for Monkey F, there were gaze–CR effects of roughly equal magnitude both above and below the diagonal. For these conditions, we selected the peak within the above-diagonal data (Fig. 4*B*, black diamonds) to maintain the temporal order of the predictive relationship that is the focus of the study (Fig. 2). At these selected points, we then computed the averaged spike–CR correlation across all cells, as in Figure 7*B* (rho_adj_), and compared it to zero by means of a *t* test. Note that for every two-cue trial type, there are two gaze–CR matrices calculated (one for each cue; Fig. 4), but only one spike–CR matrix. Therefore, to generate the data for two-cue trials in Figure 8, each spike–CR matrix is sampled at two points, one corresponding to the maximum gaze–CR effect for the lower value cue, and the other corresponding to the maximum effect for the higher value cue.

#### Mediation analysis

The objective of this analysis was to quantify the degree to which OFC activity explains the correlation between gaze allocation and reward-anticipating CRs. The term “mediation” is used in a purely statistical sense and does not by itself imply a causal relationship between the variables. As with the gaze–CR analysis (Fig. 4), a separate analysis was performed for each single-cue trial type, and two separate analyses were performed for each two-cue trial type (one each for the low- and high-value cues). Thus, nine separate mediation analyses were performed for each monkey, corresponding to the nine gaze–CR analyses shown in Figure 4.

To quantify the mediation effect attributable to a single OFC cell, we measured gaze allocation to cues, spike counts, and CR data in 500 ms bins. For every pair of time bins *x* and *y*, the following ordinary least-squares linear models were fit as follows:

(3) $$\mathrm{Model}1:\;CR_{x}∼\beta_{0}+\beta_{\mathrm{GAZE}}*\mathrm{Gaz}e_{y}$$

(4) $$\mathrm{Model}2:\;CR_{x}∼\beta_{0}+\beta_{\mathrm{GAZE}}*\mathrm{Gaz}e_{y}+\beta_{\mathrm{SPK}}*\mathrm{Spik}e_{y},$$

where CR*_x_* is the conditioned response observed within time bin *x*, Gaze*_y_* is the gaze allocation for a given cue in time bin *y*, and Spike*_y_* is the spike count from the cell in time bin *y* (observed concurrently with gaze). In this analysis, gaze allocation and CR are quantified in units of time (range, 0–500 ms), such that the regression estimate β_GAZE_ can be interpreted as the linear effect of gaze on the CR. For example, a β_GAZE_ of 0.3 would indicate that for every 1 s increase in gaze allocation, an increase of 0.3 s in CR would be expected.

If β_GAZE_ is the same magnitude in both Model 2 and Model 1, this indicates that the Spike variable explains the variance in the CR that is not attributable to the Gaze variable. In contrast, if β_GAZE_ is smaller in Model 2 than in Model 1, it indicates that the Spike variable has subsumed variance in the CR that would otherwise be accounted for by Gaze; this is evidence that Spike statistically mediates the linear association between the Gaze and CR variables.

Thus, the mediation effect for a given cell at a given pair of time bins was calculated by subtracting the β_GAZE_ estimate resulting from Model 2 from the β_GAZE_ estimate resulting from Model 1.

Nine mediation effect matrices were calculated for each OFC cell, and the median mediation effects across cells were compared with zero by means of a signed rank test. In Figure 7*D*, the heatmap shows the median matrix of mediation effects over *n* = 116 cells, measured in single “small” cue trials in Monkey K.

The mediation analysis was repeated in the subset of cells showing modulation by gaze. In this analysis, we included any cells with significant effects (*p* < 0.05 corrected) of fixation distance or the interaction variable in the analysis of single-cue trials, and any cells with significant effects (*p* < 0.05 corrected) of one of the three fixation-related variables in the analysis of two-cue trials (Table 3, rows 1-3). A total of 84 cells were included: 45 from Monkey K and 39 from Monkey F.

## Results

### Reward anticipation and gaze allocation to pavlovian cues

Two monkeys performed the pavlovian conditioning task in Figure 1. To begin a trial, monkeys briefly held their gaze on a fixation point, after which one or two visual cues appeared on the display for 4 s (Fig. 1*A*,*B*). The monkeys were free to move their eyes throughout this period; eye movements were monitored, but had no effect on the trial outcome. At the end of 4 s, a juice reward was delivered as follows: single cues resulted in a guaranteed reward of 0, 1, or 3 drops (“none”, “small”, or “large”), with reward size determined by cue color (Fig. 1*C*). The presentation of two different cues resulted in random delivery of one of the two indicated rewards, and the monkeys could not predict or influence which one would be delivered (Fig. 1*D*). Single-cue and two-cue trials were randomly interleaved; cue selection was random, as was the placement of cues on the left and right sides of the display.

When the expected reward was nonzero, monkeys made pavlovian CRs in anticipation of reward delivery, beginning ∼2 s after the onset of the cues and reaching a maximum just before reward delivery at 4 s (Fig. 1*E*). On average, the CR magnitude increased monotonically with the mean value of the cues shown (Fig. 1*F*). Importantly, the average CRs differed significantly among the three single-cue trials, indicating that the monkeys successfully learned the individual cue–reward contingencies. We therefore consider these CRs to be indicators of reward anticipation on a given trial.

The allocation of the gaze—where the monkeys looked and for how long—was quantified by the fraction of time in every trial that the monkeys fixed their gaze on each cue. Gaze allocation varied as a function of cue value, but, unlike CRs, was not monotonically dependent on cue value (Fig. 1*G*). Importantly, monkeys devoted nonzero fixation time onto “none” cues and also devoted nonzero fixation time onto the smaller of two cues shown simultaneously. Thus, the behavioral and neural effects of gaze allocation onto cues could be assessed regardless of cue value.

To summarize, monkeys were shown simple appetitive conditioned cues, either singly or in pairs. The monkeys allocated a significant portion of their gaze (overt attention) toward the cues, and performed anticipatory CRs commensurate with average cue value. The major questions of this study are whether trial-by-trial variability in gaze allocation corresponds to variability in CR magnitude (Fig. 2*A*), and whether this correlation could be mediated by the value representations expressed in single OFC neurons (Fig. 2*B*,*C*). In the next section, we document the trial-by-trial correlation between gaze allocation and CRs.

### Allocation of gaze to appetitive cues predicts trial-to-trial reward anticipation

On each trial, gaze allocation was defined as the fraction of time that the monkey spent looking at a cue (gaze <3º from cue center), and CRs were quantified according to the fraction of time that a licking response was detected. These two variables were measured in 500 ms bins at 50 ms increments during cue presentation, and the correlation between them was calculated across trials within a session for every possible pair of time bins in the trial. This yielded a measure of whether gaze allocation early in the trial was correlated with CRs later in the trial, and vice versa. Note that the CRs were only calculated at time bins with centers 2500 ms postcue or later, due to the near total lack of CRs before this time (Fig. 1*E*).

Correlations were calculated within a given session (*n* = 28 for Monkey F; *n* = 25 for Monkey K), and then averaged across sessions. Because correlation patterns differed substantially according to cue value (see below), the correlations were calculated separately for each of the six trial types shown in Figure 1, *C* and *D* (“none,” “small,” “large,” “none-small,” “none-large,” and “small-large”). Furthermore, in two-cue trial types, gaze allocation was tallied separately for each individual cue shown, permitting two separate correlation calculations to be obtained for every two-cue trial type. Therefore, for each monkey, nine total gaze–CR conditions were calculated: one for each trial type with a single cue; and two for each of the trial types with two cues.

#### Gaze–CR correlation in single trial type in a single subject

Figure 3*A* illustrates the trial-by-trial correlation between gaze allocation and pavlovian CRs in one trial type (single “small” cue) in Monkey K. The color of each pixel gives the session-wise mean correlation (rho) between the fraction of time the monkey spent looking at the cue and the fraction of time a CR was detected; correlations were calculated across all of the trials within a given session and then averaged across sessions (*n* = 25). Points above the black solid diagonal line indicate gaze data that precede (and could therefore predict) the CR data; and points below the black line indicate gaze data that follow CR data.

The highest average correlation occurred for gaze data measured at 3.05 s (*y*-axis) and CR data measured at 3.30 s (*x*-axis), with an average value of rho = 0.291 (SEM, 0.028; Table 1, b). The significant positive correlation indicates that greater time spent gazing at the cue was associated with a larger CR; and the fact that the gaze data precede the CR data at this point indicates that gaze allocation could predict the upcoming CR on a trial-by-trial basis with an ∼0.25 s temporal lag. In other words, the longer Monkey K looked at single “small” cues at ∼3 s in a trial, the more likely he was to exhibit an anticipatory CR 0.25 s later. Note that this predictive relationship was asymmetric: gaze was better able to predict CRs than the opposite, indicated by the higher correlations and greater fraction of significant pixels above the solid gray diagonal line compared with below it (Fig. 3*B*).

#### All trial types and both subjects

The gaze–CR correlations for all subjects and trial types are shown in Figure 4, using the same conventions as in Figure 3. The effects were highly variable from one condition to the next, with clear differences in the correlation patterns between subjects, and between conditions within each subject. However, despite the variability, two general patterns were evident. First, as in the example in Figure 3, the only significant correlations were positive (warm colors), meaning that longer time spent looking at cues was associated with more frequent CRs. Significant positive correlations were found for three of the nine conditions in Monkey K^d1-d3^; and seven of nine conditions in Monkey F^d7-d13^. No significant negative correlations were found. Second, gaze predicted CR performance to a greater degree than CRs predicted gaze behavior (above vs below diagonal: three of nine conditions in Monkey K^d4-d6^; and three of nine conditions in Monkey F^d14-d16^). There were no conditions in which a significant difference was found in the opposite direction (i.e., in which CR predicted gaze to a greater extent than gaze predicted CR). In summary, we identified several trial conditions in which the longer that monkeys spent looking at (attending to) reward-associated cues, the greater their subsequent anticipation of reward delivery indicated by conditioned licking responses.

### Modulation of OFC neural activity by gaze

The positive correlation between overt attention to cues and pavlovian CRs (Figs. 3, 4) must be explained by some neural mechanism that links these two behaviors. To identify this mechanism requires finding, at a minimum, neural activity related to the two behaviors of interest (Fig. 2*B*,*C*). Here, we show that OFC firing is modulated by shifts of gaze toward or away from pavlovian cues. Because the gaze–CR correlation was present in both single- and two-cue trials, it was necessary to identify the effects of gaze on OFC activity in both kinds of trials. Single-cue gaze effects were shown in a prior study and are recapitulated here using a subset of the original data (McGinty et al., 2016), consisting of those cells in which both single- and two-cue trials were tested. Data from two-cue trials are shown for the first time and are analyzed separately from single-cue trials due to differences in gaze behavior when two cues are shown rather than one (see below). A total of 116 neurons were recorded in Monkey K, and 64 neurons in Monkey F.

#### Single-cue trials

In single-cue trials, monkeys typically shifted their gaze many times during the 4 s cue presentations, fixating at various locations on the display, both on and off the cue (Fig. 1*G*,*F*; see also McGinty et al., 2016, their Fig. 2). To assess neural activity with reference to gaze location, OFC firing was measured from 100 to 300 ms after the onset of each fixation, a time window that captures the peak OFC response following shifts of gaze (McGinty et al., 2016, their Fig. S4C). This “fixation-evoked” firing was the basic unit of data for this analysis.

We fit a GLM that explained firing as a linear function of the value of the cue shown, the distance of gaze from the cue, and the value-by-distance interaction (Eq. 1). The single cells in Figure 5, *B* and *D*, illustrate the encoding of all three variables, as follows: firing was greatest for fixations near to the cue (distance encoding), and was monotonically related to the volume of juice reward (value encoding). Critically, the effect of value was greatest for near-to-cue fixations, which constitutes an interaction between the value and distance effects. At the population level, large portions of neurons were significantly modulated by cue value (47.8% and 33.9% with GLM effects at *p* < 0.05, uncorrected and corrected for multiple comparisons, respectively) and fixation distance from the cue (38.9% and 23.9%), and a smaller portion were modulated by the interaction term (25.0% and 7.2%). As was the case in the prior study, the distributions of regression estimates were continuous and unimodal for all three variables (for an illustration, see McGinty et al., 2016, their Fig. 5*B*). The mean regression estimate for the distance effect was significantly less than zero (−0.0096; SEM, 0.0020; *p* = 4.3 * 10^−6^)^e^, indicating that near-cue fixations elicited greater overall firing than fixations away; and the mean value estimate was not significantly different from zero (0.032; SEM, 0.050^f^), indicating that neurons were as equally likely to increase firing with cue value (Fig. 5*B*) as they were to decrease firing (Fig. 5*D*). Many neurons had more than one significant effect, indicated in the Venn diagram in Figure 6*A* (compare McGinty et al., 2016, their Fig. 5*A*).

To determine whether the same neurons tended to encode multiple variables, we took the absolute values of the regression estimates and then calculated the correlations between them. Positive correlations indicate that cells with nonzero estimates in one variable tend to have nonzero estimates in the other, whereas negative correlations indicate that cells with nonzero estimates for one variable tend to have close-to-zero estimates in the other. In our data, all pairwise correlations between the regression estimates were significantly positive, as follows: between the value and distance estimates, the correlation was *r* = 0.271 (*p* = 0.0002)^t^; between value and the interaction term, the correlation was *r* = 0.254 (*p* = 0.0005)^u^; and between distance and the interaction term the correlation was *r* = 0.292 (*p* = 7 × 10^−5^)^v^. These positive correlations indicate that the same neurons tended to encode multiple variables more often than expected by chance.

#### Two-cue trials

In two-cue trials, monkeys shifted their gaze throughout cue presentation. However, unlike in single-cue trials, the majority of fixations were directed onto the cues (Fig. 1*G*), leaving too few off-cue fixations to assess the effects of gaze distance. The analysis therefore used only firing evoked by on-cue fixations, again within a 100–300 ms window after each. On-cue fixations are parameterized by the values of the fixated and nonfixated cues, so that gaze effects can be assessed by the degree to which firing is modulated by either of these variables. In addition, we assessed modulation by cue value in a non-gaze-dependent manner, given that during single-cue trials we found cells that encode only cue values with no effect of gaze distance (Fig 6*A*; McGinty et al., 2016).

To identify gaze-modulated cells in two-cue trials, we began by fitting three GLMs per cell, each with a single variable that explained firing on the basis of cue values and the location of fixation. The first GLM explained firing according to the value of the cue targeted in a given fixation (“fixated value”). Two examples of cells modulated by fixated values are shown in Figure 5. The cell in Figure 5*C* fires more for fixations onto the higher value of the two cues shown, whereas the cell in Figure 5*E* fires more for the lower of the two values. The second GLM explained firing as a function of the value of the nonfixated target (“nonfixated value”); and the third GLM explained firing as a function of the value difference between the fixated and nonfixated target (“relative value”; see Materials and Methods). In all, 36.7% (*n* = 66) of OFC cells showed significant effects (corrected for multiple comparisons) of at least one of the three GLMs described above, with the large majority of these showing significant modulation by fixated value (Fig. 6*B*). Therefore, just as in single-cue trials, a substantial portion of OFC neurons have value signals that are modulated by gaze when two cues are shown.

While the primary objective of this analysis was to identify gaze-modulated cells in two-cue trials, additional analyses below provide a more comprehensive view of the variables encoded in this phase of the task. Specifically, we assess the following: encoding of non-gaze-dependent value variables; the mixture of variables encoded in single neurons; and the consistency of encoding between the single- and two-cue phases of the task.

First, to identify OFC cells modulated only by value (with no effect of gaze), we fit three additional GLMs with single variables that depended on the value of the visible cues, but not on which cue was targeted by a given fixation. These were the following: the maximum value of the two cues; the minimum value of the two cues; and the mean value of the two cues. In all, 33.9% (*n* = 61) of OFC cells had significant effects (*p* < 0.05 corrected) of at least one of these three “value-only” variables (Fig. 6*C*).

In the single-cue analysis, the variables of interest were mixed at the single-cell level (i.e., many single neurons were modulated by more than one variable). In the two-cue data, it was not possible to fit all six variables in the same GLM due to linear dependency among the regressors (see Materials and Methods). Therefore, to quantify mixed encoding of gaze-dependent and value-only variables, we used a competitive modeling approach. First, we identified all cells that had a significant effect in at least one of the six single-variable GLMs above (*n* = 77, *p* < 0.05 corrected). Then, in these cells we also fit a set of two-variable GLMs using different combinations of the six variables (Table 3, Models 7–12). Finally, we calculated the goodness of fit (AIC) for all single-variable and two-variable models, and identified which model provided the best fit for a given cell. The results are shown in Table 3 and Figure 6, *B* and *C*. The model that provided the best fit for the most cells was the two-variable model including the fixated value and the maximum value (*n* = 20, 11% of all cells), which is consistent with these two variables producing the most significant effects when fit individually (Fig. 6*B*,*C*). In all, 40 cells (22%) were best fit by a two-variable model that included one gaze-related variable and one value-only variable. Thus, as in single-cue trials, OFC neurons encode a mixture of task variables when two cues are shown.

Finally, we found that the same cells tended to be modulated in both the single-cue and two-cue task phases, as follows: 83 cells were modulated by at least one of the three single-cue analysis variables (*p* < 0.05 corrected); 77 cells were modulated by at least one of the six two-cue analysis variables; and 53 cells were modulated by at least one variable in each task phase, which was significantly greater than expected by chance (35.5 expected^g^). This was also true when considering neurons with gaze modulation, as follows: 48 cells were significantly modulated by either fixation distance or the distance-by-value interaction in the single-cue analysis; 66 cells were modulated by at least one of the three gaze-dependent variables in the two-cue analyses; and 30 cells were gaze modulated in both task phases, significantly more than expected by chance (17.6 expected^h^). Finally, the sign of value modulation was consistent across the population: assessed in all 180 cells, the regression estimates for values obtained in the single-cue analysis were highly correlated with the regression estimates for fixated value (*r* = 0.55^i^), maximum value (*r* = 0.72^j^), and mean value (0.69^k^) obtained in the two-cue analysis.

#### Summary

Shifts of gaze during the presentation of pavlovian conditioned cues influenced the firing OFC neurons in a 100–300 ms window following the onset of each fixation. When single cues were presented, many OFC cells encoded the distance of gaze from the cue, or expressed value signals that were modulated according to gaze distance. When two cues were presented, a portion of OFC cells encoded the value of the cue fixated at any given moment, the value of the other (nonfixated) cue, or both of these variables (relative value).

### OFC neural activity does not predict reward anticipation

Our central hypothesis is that shifts of gaze influence pavlovian CRs through the modulation of OFC neural activity (Fig. 2). Above, we establish the first part of this mediation relationship (gaze shifts modulate OFC activity; Figs. 5, 6). Here, we test the second arm of the mediation relationship, between OFC neural activity and CRs. To preview the results in brief, we find that, on average, OFC activity is only weakly predictive of CRs and appears insufficient to act in a mediating role.

As in the gaze–CR analysis, we measured CRs (the fraction of time a licking response was detected) and OFC activity from individual cells (spike count) in 500 ms bins. We then calculated the across-trial correlation between spike count and CRs, using an unsigned correlation metric (rho_adj_) that takes a positive value regardless of whether spiking increases or decreases with respect to the CR, thereby placing all OFC cells on the same scale (see Materials and Methods). This was done for every possible pair of time bins, yielding a matrix of spike–CR correlations across different time points in the trial. A separate spike–CR correlation matrix was created for each of the six trial types shown in Figure 1, *C* and *D*. These were then averaged across all recorded cells.

The heatmap in Figure 7*B* shows the spike–CR correlation matrix for single “small” reward trials in Monkey K, averaged across 116 cells. Unlike the gaze–CR correlation for these trials (Fig. 7*A*), the spike–CR correlations are not statistically different from zero and show no overall temporal pattern. In other words, within this example data, gaze explains the variance in CRs, but concurrently observed neural activity does not. Intuitively, the weak correlations in Figure 7*B* and the mismatch with respect to Figure 7*A* are evidence against a mediation relationship. We performed two analyses to quantify this intuition. First, we identified the time bins with the strongest gaze–CR correlation and then asked whether the spike–CR correlation at this point was significantly above zero. In Figure 7*A*, this point is marked with a gray square, at *x* = 3.30 s and *y* = 3.05 s. The mean gaze–CR correlation at this point is rho = 0.291 (SEM, 0.028)^b^. In contrast, the spike–CR correlation at this point is only rho_adj_ = 0.012 (SEM, 0.011), and is not significantly above zero (*p* = 0.26 by *t* test)^n^. In other words, even when the predictive effect of gaze was strongest, there was no corresponding predictive effect in the OFC spiking data. We repeated this analysis in all trial types for both monkeys, selecting the time points at which the predictive correlation of gaze for CRs was maximal (see Materials and Methods). As shown by the white bars in Figure 8, the average spike–CR correlations were weak for all of the selected points (Table 1, s3–s11 and s18–s12); in only two instances was the spike–CR effect significantly above zero (Table 1, s7 and s19). Thus, there was very little evidence that OFC mediated the predictive relationship between gaze and CRs, even when this effect was maximal within a given trial type.

To confirm this result, we directly quantified the mediating effect of OFC with a mediation model and expanded the scope of the analysis to consider all time bins (not just the gaze–CR maximum). For the example data (“small” trials for monkey K), the results are shown in the heatmap in Figure 7*D*. Positive values indicate evidence in favor of a mediation relationship (see Materials and Methods). The mediation effect at the maximum gaze–CR point (Fig. 7*D*, black square) was not significantly above zero (median, 2.7 × 10^−4^; SEM, 2.8 * 10^−3^)^q^. We repeated this analysis in all trial types in both monkeys and found similar weak effects (data not shown), with the strongest effect occurring in single “small” cue trials in Monkey F (median, 0.0064; SEM, 0.0049)^r^. There were no significant differences in mediation effects between single-unit and multiunit responses (data not shown).

Moreover, mediation effects were weak at virtually all time points. In the example heatmap (Fig. 7*D*), very few points show significant effects surpassing even an uncorrected threshold of *p* < 0.001^p^, and most of these lie well below the diagonal and are therefore inconsistent with the mediation hypothesis, which dictates that gaze (and spiking) temporally precede CR behavior (Fig. 2). The heatmap of mediation effects in Figure 7*D* was representative of the results from other trial types in both monkeys (data not shown); that is, mediation effects were weak overall, and only a tiny fraction of points showed effects that were significantly distinguishable from zero.

Our central hypothesis (Fig. 2) holds that mediation effects should be evident only in cells that are modulated by gaze, which we identify in Figure 6, *A* and *B*. However, even when we averaged the mediation effects of only gaze-modulated cells (*n* = 45 for Monkey K; *n* = 39 for Monkey F; see Materials and Methods), the results were the same: no significant mediation at the points with the strongest gaze–CR effects, as well as weak mediation effects overall (data not shown).

In summary, while we found evidence that shifts of gaze toward pavlovian cues positively predicted reward-anticipating CRs, we found no evidence that CRs could be predicted by concurrently observed OFC firing and, by extension, no evidence that OFC firing participates in the neural mechanisms that link attention and CRs.

## Discussion

Neurons in the primate OFC represent the value of appetitive and aversive pavlovian conditioned stimuli, suggesting a role for OFC in the subjective value signals that ultimately inform pavlovian responding. Our prior work showed that OFC value signals were modulated by moment-to-moment shifts of gaze (overt attention) toward pavlovian cues but left open the question of how this attentional modulation ultimately influences behavior. This was the core question of the current study, a timely issue in light of the clear role of attentional shifts in economic decisions (Krajbich et al., 2010; Towal et al., 2013; Vaidya and Fellows, 2015), another form of appetitive motivated behavior. Our results therefore inform the larger effort to untangle the complex relationships among attention, neural value signals, and behavior.

In monkeys performing an appetitive pavlovian conditioning task, gaze allocation positively predicted CRs: the longer the monkeys spent looking at a conditioned cue, the greater the likelihood that they would perform a conditioned licking response later in the trial—though this effect differed between monkeys and between trial types within a monkey (Fig. 4). OFC neural activity in this task was modulated by shifts of gaze, both for single cues presented alone, as reported previously (McGinty et al., 2016), and for cues presented in pairs. However, OFC activity did not predict conditioned licking responses on a trial-by-trial basis, and as a result we found no evidence that OFC firing could mediate the effects of attention on conditioned responses. Below, we discuss each of these findings in depth.

### Attention predicts conditioned responses performed in anticipation of reward

When it was present, the correlation between gaze and CR was always positive, meaning that more gaze devoted to a cue was associated with more frequent CRs. In addition, gaze predicted subsequent CRs to a greater extent than the opposite. Together, these suggest that gaze enhances the subjective value of pavlovian cues, similar to the effects on objects offered during economic choice. However, this conclusion comes with several important caveats. First, we did not directly manipulate gaze and so cannot conclude with certainty that it has a causal effect on CRs. Second, the correlations were not uniform in both subjects: whereas Monkey F had consistent effects for both “small” and “large” value cues, effects in Monkey K were prominent for “small” cues and weak or absent for “large” cues. In addition, for single-cue trials in Monkey F, CR data appeared to predict gaze behavior to a similar extent as gaze predicted CRs. However, despite these differences, the following two key patterns are present in both subjects: the positive sign of the correlation, and the overall greater predictive effect of gaze for subsequent CRs.

The source of variability in the gaze–CR correlations is not immediately clear, given the inconsistency between the two subjects, in particular, the negligible effects for “large” value cues in Monkey K relative to those in Monkey F. One possibility is that for Monkey K, the largest available reward is in essence a “jackpot” for which the subjective value is maximal and therefore inelastic. This is consistent with observations in humans that larger rewards are subject to shallower discount functions than smaller rewards (Green et al., 1999).

The inconsistency of gaze–CR effects also makes it difficult to specify the nature of the neural mechanism that links attention and reward anticipation. In economic choice, computational models support a multiplicative mechanism in which gaze effects increase as a function of the value of the attended item (Krajbich et al., 2010; Towal et al., 2013; Vaidya and Fellows, 2015). The data for Monkey K are inconsistent with a multiplicative mechanism, with virtually no gaze effects for the highest value cue. Our results also appear to rule out a simple additive mechanism (Cavanagh et al., 2014), because the overall weak effects on “none” value cues suggest that gaze must interact with, rather than simply amplify, neural value representations that underlie CRs. Additionally, gaze effects in both monkeys appear to be sensitive to whether cues are presented alone or in pairs, as follows: Monkey K shows a positive gaze–CR effect for “small” cues presented alone, but not when presented alongside “large” value cues; and Monkey F shows a positive gaze–CR effect for “none” cues presented alone, but not alongside another cue. This suggests that gaze effects may depend on the relative cue value.

In summary, attending to pavlovian cues appears to enhance their subjective value. While broadly similar to attentional effects in economic choice, the underlying mechanisms may differ. Additional experiments using a greater variety of reward values and a larger number of subjects may be necessary to resolve this question.

### Attention modulates OFC value signals

OFC value signals for single cues are modulated by overt shifts of attention toward or away from those cues (McGinty et al., 2016). The current study extends these findings to gaze shifts between two items of different value. In general, the effects of gaze were consistent in both single-cue and two-cue contexts. In both contexts, many OFC cells represented cue value in some form, but only a subset were also modulated by shifts of gaze. In two-cue trials, attended items were preferentially represented, indicated by the fact that the fixated value variable was represented by a greater fraction of cells than the nonfixated value (Fig. 6*B*). This is consistent with gaze effects in single-cue trials, where OFC neurons express a stronger distinction between the cue values when the monkeys gaze toward the cues, illustrated in the example cells in this study (Fig 5*B*,*D*), and in cell-averaged data in the study by McGinty et al. (2016, their Fig. 5*D*,*E*). The preferential representation of attended over unattended items is also consistent with the effects of covert shifts of attention, obtained under similar pavlovian-like conditions (Xie et al., 2018). Our findings are also consistent with those of Hunt et al. (2018), who report OFC value signals that primarily reflect the fixated object during a decision-making task. In contrast, one recent report (Rich and Wallis, 2016) showed no effect of fixation on OFC value signals; this may be attributable to differences in task design, for example, the fact that fixations were required to identify the choice targets in the study by Hunt et al. (2018), but not in that by Rich and Wallis (2016).

In summary, whether one or two value-associated objects are present, a substantial portion of the value representation of the OFC is modulated by shifts of gaze during natural free viewing. An open question is whether the effects of overt attention and those of covert attention are two facets of the same common neural mechanism, or result from two distinct neural mechanisms under different experimental contexts (i.e., free viewing vs enforced fixation).

### Potential mechanisms for attentional modulation of conditioned responses

OFC activity was almost entirely uncorrelated with the performance of CRs, meaning there is no evidence that the OFC mediates the predictive relations between gaze and CRs. This negative finding is at odds with the observations of Morrison and Salzman (2009), who found that the responses of OFC neurons to pavlovian cues could indeed predict the performance of conditioned responses on a trial-by-trial basis. More generally, lesion evidence implicates the OFC and nearby ventral and medial prefrontal areas in conditioned autonomic responses, including changes in heart rate (Reekie et al., 2008) and in pupil constriction in response to conditioned cues (Rudebeck et al., 2014; Hwang et al., 2018). This is consistent with anatomic evidence showing projections from these areas to lateral hypothalamic regions that innervate autonomic output centers in the brain and spinal cord (Barbas et al., 2003).

The discrepancy between our findings and those of Morrison and Salzman (2009) may be explained by a difference in task design. First, in Morrison and Salzman (2009) the visual cues appeared only briefly (∼300 ms), followed by a trace interval of 1.5 s with no stimuli present. Second, both appetitive and aversive unconditioned stimuli were used (juice reward and an air puff), corresponding to two distinct CRs. Thus, to perform the task optimally, the monkeys were required to remember the conditioned cue over the trace interval and to perform the correct response in anticipation of the associated outcome. In contrast, our task had no mnemonic requirement and only one possible outcome. It is possible that the greater working memory and behavioral demands of the task used by Morrison and Salzman (2009) may have required greater recruitment of prefrontal circuitry and, therefore, produced a measurable correlation between OFC activity and behavior.

Negative findings are not in themselves evidence of no effect. Two follow-up experiments could potentially clarify these findings. First, simultaneous recording from multiple neurons would allow for less noisy estimates of subjective value signals on single trials and, therefore, less noisy estimates of the mediation effects. This approach may be particularly appropriate for OFC, where individual cells appear to be poor estimators of underlying value variables due to high within-cell noise and low across-cell correlation (Conen and Padoa-Schioppa, 2015). Second, direct manipulation of OFC neurons would establish whether gaze–CR predictions depend on normal OFC function.

If the negative mediation findings are indeed reliable, then regions other than OFC must form the neural mechanism linking gaze and reward anticipation. These may be regions that are both involved in consummatory oromotor movements (i.e., the licking response measured in this study) and are also subject to attentional modulation. One candidate region is the ventral striatum (VS). Although oromotor responses to pleasant and unpleasant tastes do not require the VS (or any circuitry above the midbrain; Grill and Norgren, 1978), they can be influenced by stimulation of the VS (Krause et al., 2010) and by pharmacological manipulations, especially of dopamine and opioid receptors in the rostral shell of the nucleus accumbens (Castro et al., 2015). VS manipulations also modulate the incentive value of pavlovian conditioned stimuli (Corbit et al., 2001) and influence reward-seeking behavior in response to those stimuli (Nicola, 2010; Hoffmann and Nicola, 2014). In a human imaging study by Lim et al. (2011), value representations evident in the VS BOLD signal were modulated by gaze shifts between visual objects of differing values, similar to the modulation exhibited by OFC neurons in this study. Thus, the VS exhibits both attention-modulated value signals and exerts top–down control over appetitive oromotor responses, making it a candidate region for mediating the effects of attention on reward anticipation that we observed in this study.

Other candidates are regions projecting to the VS, particularly the ventromedial prefrontal cortex (vmPFC), which projects to the shell of the nucleus accumbens (Heilbronner et al., 2016) and also express gaze-modulated value signals in humans (Lim et al., 2011). In contrast, the region recorded in this study, Walker’s area 13, projects primarily to the ventromedial caudate nucleus and accumbens core, with only weak projections to the accumbens shell (Haber et al., 1995). The direct connections of Area 13 with the vmPFC are also sparse (Carmichael and Price, 1996; Ongür and Price, 2000). Thus, the overall limited connectivity between the recorded region and putative oromotor output centers may account for the very weak correlations we observed between neural activity and CRs. By this logic, we predict that neurons in vmPFC should exhibit stronger correlations with conditioned licking than neurons in OFC. Apart from the VS and vmPFC (which are specifically implicated in oromotor responses), other potential regions of interest include the amygdala and insular cortex, owing to their involvement in other forms of conditioned appetitive behavior, such as autoshaping (Cardinal et al., 2002; Nasser et al., 2018), and the anterior cingulate cortex, owing to its role in motivation more generally (Cohen et al., 1999; Darby et al., 2018).

### Implications for decision-making and other motivated behaviors

Overt shifts of attention influence another important form of motivated behavior, economic choice. During decision-making, choice is biased in favor of the item fixated on (attended to) first in a given trial, as well as toward the item that received the largest overall portion of the total fixation time before the choice (Krajbich et al., 2010; Towal et al., 2013; Vaidya and Fellows, 2015; Tavares et al., 2017). This effect is well explained by serial sampling models in which fixation biases the accumulation of evidence in favor of whichever item is attended at any given moment (Krajbich et al., 2010; Tavares et al., 2017). Neurons that preferentially encode the value of attended stimuli—like those reported here—would be an important element of such a mechanism, providing input to downstream circuitry (presumably proximal to motor outputs) in which the evidence accumulation takes place. In theory, a similar mechanism could underlie gaze effects on pavlovian responses. However, our results suggest that the OFC value signals do not perform this function in pavlovian contexts.

These results therefore suggest two related questions that must be addressed in future work. The first concerns the precise locus of attention-modulated value signals that ultimately influence behavior. The OFC appears to not be involved in gaze modulation of pavlovian responses, but its role in gaze effects on economic choice is unclear. For example, Vaidya and Fellows (2015) show in humans that that large bilateral lesions of the OFC, vmPFC, and underlying white matter do not affect choice biases attributable to gaze. While this is consistent with the negative findings we report here, it must be confirmed with neural recordings and more precise causal manipulations in the appropriate animal model. And the second question concerns the extent to which the attentional effects on choice and pavlovian responses are attributable to common neural substrates. As we note above, the inconsistent gaze modulation for high value cues would not be expected according to computational models of choice, suggesting at least one major mechanistic difference between choice and pavlovian contexts.

## Synthesis

Reviewing Editor: Mark Baxter, Mount Sinai School of Medicine Decisions are customarily a result of the Reviewing Editor and the peer reviewers coming together and discussing their recommendations until a consensus is reached. When revisions are invited, a fact-based synthesis statement explaining their decision and outlining what is needed to prepare a revision will be listed below. The following reviewer(s) agreed to reveal their identity: Benjamin Hayden.

The reviewers noted with approval the insights provided by the reanalysis of the data and the care with which analyses controlled for other possible explanations of the failure to find a relationship between OFC activity and value. The following are suggestions for clarification -

Methods -

Lines 115-119 “Using aseptic techniques, they were implanted with an MR-compatible head holder, and subsequently with a recording chamber (Crist Instruments, Hagerstown, MD); a craniotomy was also performed to allow access to the OFC. Data were collected while the monkeys were head-restrained and seated ~57 cm from a fronto-parallel CRT monitor displaying the task stimuli.” The authors should re-state that these procedures were done under full anesthesia and with appropriate antibiotics, analgesics etc. This is a key detail for publication as if they were not, the research cannot be published at eNeuro as it would violate requirements for research with non-human primates.

Lines 169-172 “In a prior report from this data set (McGinty et al., 2016), we examined the effects of cue-reward ‘reversals’ on some OFC cells. Those cells are also used here; however, for a given cell we use only the pre- or post-reversal data (never both) according to whichever segment of the data had more trials.” While I very much doubt that it will change the results, the author should state how many cells were included with pre and post reversal phases and also account for any differences in the overall results when either the pre or post reversal phase was used in the analyses of the relationship between CRs and OFC neural activity.

Line 229-233 “Among the single units, some were isolated during the learning phase of the task (see above) and were selected for subsequent recording because they showed an apparent increase or decrease in firing during cue presentation; the remainder of the single units, and all multi-unit signals, were recorded without any prior observation of their activity during task performance.” While it is great that the author is being honest about unit isolation, this statement means that the selection of units was not uniform. What was the proportion of units that were recorded which were selected based on their functional properties? If these are removed from the analyses does it alter the main findings? I realize that this may be hard to quantify but confidence intervals or equivalent on this point would be useful.

Results -

How was the epoch of 100-300 ms selected?

“The mean regression estimate for the distance effect was negative” but was it significantly negative? (not sure, looking at SEM probably is). Either way, should be mentioned and justified.

For the Venn diagram in Figure 6.... I have found that this approach to cell classification is somewhat non-ideal ... it imposes categories on what are fundamentally continuous data (unless you have evidence the data are categorical, in which case this is the best best approach). This approach throws away a lot of information and, while conservative, does lead to systematic biases in interpreting population responding. It can also lead to false positives, depending on your hypotheses. (Blanchard, Piantadosi, and Hayden, 2018 provides additional detail.). Anyway, because of that it's less misleading to compute an index of responding (e.g. the linear term of the regression weight) and compare those. Thus for example, the important question, which the reader can kind of get from the Venn diagram, is, are the unsigned betas for value and distance correlated? A positive correlation indicates cells from from a single larger pool and if they are anti-correlated, they likely come from two separate pools.

“(47.8 and 33.9% with GLM effects at p<0.05, uncorrected and corrected, respectively)” what do corrected and uncorrected refer to here (for readers who dont want to hunt through the methods) - is this multiple comparison correction?

“In all 36.7% (n=66) OFC cells showed significant effects of at least one of the three GLMs described above” is this corrected for multiple comparisons?

Line 627 - does the use of rho instead of r/r-squared mean it was a spearman correlation? that's fine if there are outliers but even so, it's a less sensitive metric than pearson. thats important when considering a null result as the main result.

Line 644 - one can also perform a fisher z-to-r to show that the second correlation is itself significantly lower than the first.

Line 654- but not different width of time bins, right?

Discussion -

Lines 683-685 “Neurons in the primate OFC represent the value of appetitive and aversive Pavlovian conditioned stimuli, and can predict the performance of a Pavlovian conditioned response (Morrison and Salzman, 2009).” I found it a little odd that the author started the discussion section this statement when their findings presented in the prior results section essentially refute this point. Later in the discussion they go into detail as to why these two studies differed but omitted a major difference: Morrison and Salzman focused heavily on the period where monkeys were learning. By contrast, here the author threw out trials where monkeys were not showing discriminatory CRs. So, I have a couple of questions based on this:

1) how many trials on average did it take the monkeys to exhibit “learning” as assessed by CRs in the present study? On line 168 the author quotes 60-100 learning trials, but no more information on this is given.

2) Was there any relationship between the emergence of learning related CRs and gaze patterns? In that if learning occurred faster in a session was this associated with a higher correlation between CRs and eye movements? Alternatively, if the learning period is analyzed using a sliding window approach across trials does discriminatory looking behavior precede the onset of discriminatory CRs?

3) How do the main physiology results compare if the learning period is analyzed on its own (although I realize that there may not be enough statistical power to do this) or this period is included in the main analysis?

4) Are their CRs that the author thinks the OFC activity might be more related to? They briefly touch on autonomic responses but don't elaborate much more on this. They might want to note the other work showing that OFC lesions affect learning related autonomic responses and behavior (Reekie et al., PNAS, 2008).

In the discussion about how other areas outside of OFC could be involved in anticipatory licking, a motoric conditioned approach response, the author might want to have a look at the autoshaping literature in rodents. For instance, see Cardinal et al., Behavioral Neuroscience (2002). Another place to look could be the literature on volitional control of action/abulia which implicates the anterior cingulate cortex.

## Author Response

Dear Dr. Baxter,

I am pleased to submit to eNeuro the revised version of our manuscript, “Overt attention towards appetitive cues enhances their subjective value, independent of orbitofrontal cortex activity”.

I have addressed all of the reviewers' comments, and included all of the requested new analyses, with the exception of one analysis for which the underlying data were unavailable (see point 14 below). The new analyses include one new figure which I have reproduced below, but not included in the main manuscript, given that is a negative, confirmatory result; I am happy to upload this as extended data if you think it appropriate.

Please do not hesitate to contact me if any additional changes or clarifications are called for. Once again, my thanks to you and the reviewers for the timely review.

Yours,

Vince McGinty

Response to reviews

1. Lines 116-120 “Using aseptic techniques, they were implanted with an MR-compatible head holder, and subsequently with a recording chamber (Crist Instruments, Hagerstown, MD); a craniotomy was also performed to allow access to the OFC. Data were collected while the monkeys were head-restrained and seated ~57 cm from a fronto-parallel CRT monitor displaying the task stimuli.” The authors should re-state that these procedures were done under full anesthesia and with appropriate antibiotics, analgesics etc. This is a key detail for publication as if they were not, the research cannot be published at eNeuro as it would violate requirements for research with non-human primates.

The text has been changed as requested. See lines 115-120 of the revised manuscript:

“They were implanted with an MR-compatible head holder, and subsequently with a recording chamber (Crist Instruments, Hagerstown, MD); a craniotomy was also performed to allow access to the OFC. All surgical procedures were performed under full surgical anesthesia using aseptic techniques and instruments, with analgesics and antibiotics administered pre-, intra- and/or post-operatively as appropriate.”

2. Lines 169-172 “In a prior report from this data set (McGinty et al., 2016), we examined the effects of cue-reward ‘reversals’ on some OFC cells. Those cells are also used here; however, for a given cell we use only the pre- or post-reversal data (never both) according to whichever segment of the data had more trials.” While I very much doubt that it will change the results, the author should state how many cells were included with pre and post reversal phases and also account for any differences in the overall results when either the pre or post reversal phase was used in the analyses of the relationship between CRs and OFC neural activity.

Information about reversal data has been added in lines 176-179 of the revised manuscript:

“In Monkey F, all cells used pre-reversal data only (n=64), and in Monkey K, 69 out of 116 cells used pre-reversal data and the remaining 47 used post-reversal data. The results for Monkey K did not differ between the cells using pre- and post-reversal data (not shown).”

3. Line 229-233 “Among the single units, some were isolated during the learning phase of the task (see above) and were selected for subsequent recording because they showed an apparent increase or decrease in firing during cue presentation; the remainder of the single units, and all multi-unit signals, were recorded without any prior observation of their activity during task performance.” While it is great that the author is being honest about unit isolation, this statement means that the selection of units was not uniform. What was the proportion of units that were recorded which were selected based on their functional properties? If these are removed from the analyses does it alter the main findings? I realize that this may be hard to quantify but confidence intervals or equivalent on this point would be useful.

We agree that unit selection is an important factor in interpreting neural results. However, we did not routinely note which single units were ‘selected’ and which were not, and so the data fully address this question. That said, the fact that multi-unit responses were always ‘non-selected’ means that they represent an unbiased sample of neural activity. Therefore, if the multi-unit results show no systematic differences from the single unit results, it would suggest that the selection procedure did not appreciably bias the findings.

To test this, we calculated mediation effects at the gaze-CR peak. This is the same procedure as the mediation analysis in the main text (see lines [681-684], the black square in Figure 7D, and rows q and r of Table 1), except that it was performed separately for single and multi-unit data. The boxplots below show the results of this analysis for all nine conditions in both monkeys. Dots show outliers, and the triangles give 95% confidence intervals about the median, defined as +/- 1.57/sqrt(n) (see Chambers JM, ‘Graphical Methods for Data Analysis, pp60-63, 1983).

Within each condition, we compared single unit to multi-unit data using a rank sum test. None of the comparisons reached statistical significance after correction for multiple comparisons, though one comparison was near significance after correction, as shown in the graph.

In sum, the data obtained from unbiased multi-unit responses appear to be no different than the data obtained from single units, and support the overall conclusion of a negative mediation effect. While this falls short of an explicit comparison between ‘selected’ and ‘non-selected’ responses, the lack of any systematic differences between single and multi-unit responses suggests argues against the existence of substantial selection effects.

We are happy to include these boxplots as extended data, if the reviewers or editor think they might be of sufficient interest to the reader. Our preference would be to only mention these results, and so the following text has been added at line 685:

“There were no significant differences in mediation effects between single units and multi-unit responses (not shown).”

4. How was the epoch of 100-300 ms selected?

The following text has been added to lines 298 and 539:

“This time window captures the peak fixation-evoked response in gaze-responsive OFC cells, as illustrated in McGinty et al. (2016), their Figure S4C.”

“ ... a time window that captures the peak OFC response following shifts of gaze (see McGinty et al. (2016), their Figure S4C).”

5. “The mean regression estimate for the distance effect was negative” but was it significantly negative? (not sure, looking at SEM probably is). Either way, should be mentioned and justified.

The text in line 553 has been changed to:

“The mean regression estimate for the distance effect was significantly less than zero (-0.0096, SEM 0.0020, p = 4.3*10-6) ...”

6. For the Venn diagram in Figure 6.... I have found that this approach to cell classification is somewhat non-ideal ... it imposes categories on what are fundamentally continuous data (unless you have evidence the data are categorical, in which case this is the best best approach). This approach throws away a lot of information and, while conservative, does lead to systematic biases in interpreting population responding. It can also lead to false positives, depending on your hypotheses. (Blanchard, Piantadosi, and Hayden, 2018 provides additional detail.).

We agree that the underlying data are continuous, and that the Venn diagrams tend to obscure this fact. In the prior study we show in detail the continuous nature of the data with scatter plots and marginal histograms (see McGinty et al 2016, Figure 5B and C), and for that reason we did not do so here. The text on line 551 has been updated as follows, to ensure no confusion on this point:

“As was the case in the prior study, the distributions of regression estimates were continuous and unimodal for all three variables (for an illustration see McGinty et al. 2016, their Figure 5B.)”

7. Anyway, because of that it's less misleading to compute an index of responding (e.g. the linear term of the regression weight) and compare those. Thus for example, the important question, which the reader can kind of get from the Venn diagram, is, are the unsigned betas for value and distance correlated? A positive correlation indicates cells from from a single larger pool and if they are anti-correlated, they likely come from two separate pools.

Thank you for this suggestion. We have added the following results, starting at line 560:

“To determine whether the same neurons tended to encode multiple variables, we took the absolute values of the regression estimates, and then calculated the correlations between them. Positive correlations indicate that cells with non-zero estimates in one variable tend to have non-zero estimates in the other, whereas negative correlations indicate that cells with non-zero estimates for one variable tend to have close-to-zero estimates in the other. In our data, all pairwise correlations between the regression estimates were significantly positive: Between the value and distance estimates, the correlation was r = 0.271 (p = 0.0002)t; between value and the interaction term r = 0.254 (p = 0.0005)u; and between distance and the interaction term r = 0.292 (p = 7x10-5)v. These positive correlations indicate that the same neurons tended to encode multiple variables more often than expected by chance.”

8. “(47.8 and 33.9% with GLM effects at p<0.05, uncorrected and corrected, respectively)” what do corrected and uncorrected refer to here (for readers who dont want to hunt through the methods) - is this multiple comparison correction?

The text on line 548 been changed, as follows:

“ . . . (47.8 and 33.9% with GLM effects at p<0.05, uncorrected and corrected for multiple comparisons, respectively) . . .”

9. “In all 36.7% (n=66) OFC cells showed significant effects of at least one of the three GLMs described above” is this corrected for multiple comparisons?

The text on lines 589 has been changed, as follows:

“In all 36.7% (n=66) OFC cells showed significant effects (corrected for multiple comparisons) of at least one . . .”

10. Line 627 - does the use of rho instead of r/r-squared mean it was a spearman correlation? that's fine if there are outliers but even so, it's a less sensitive metric than pearson. thats important when considering a null result as the main result.

That is correct. The relevant text in the Methods now reads as follows, starting at line 260:

“The correlation statistic was Spearman's rho, a non-parametric, outlier-resistant, ranks-based measure of association. It is preferred over Pearson's r because the data were not normally distributed: Measurements of gaze and CR durations were bounded by the 500ms bin size, and had a Bernoulli-like distribution; and the spiking data (below) naturally took on a Poisson-like distribution. ”

11. Line 644 - one can also perform a fisher z-to-r to show that the second correlation is itself significantly lower than the first.

While this analysis does have some intuitive appeal, in our opinion it would not add to the findings in a meaningful way. First, conceptually, comparing these two effects (gaze-CR and spike-CR) is not the primary objective. Rather, the objective is to assess whether any reliable spike-CR correlation can be detected, and, critically, whether the spike-CR data are consistent with a mediating influence between gaze and CRs. For example, one could imagine a spike-CR effect that is significantly less than the gaze-CR effect, but that is still sufficiently above zero to support mediation. In this case, the mediation result would be the key finding, whereas the difference in correlations would merely be incidental. For that reason, we did not formally compare the two correlations.

Second, on a technical note, the spike-CR correlation is calculated using rho_adj, a modified version of rho (see Eqn 2, line 357), and so it's unclear that a Fisher transformation could be applied in the same way as it would be for a standard rho or r.

12. Line 654- but not different width of time bins, right?

Correct.

13. Lines 683-685 “Neurons in the primate OFC represent the value of appetitive and aversive Pavlovian conditioned stimuli, and can predict the performance of a Pavlovian conditioned response (Morrison and Salzman, 2009).” I found it a little odd that the author started the discussion section this statement when their findings presented in the prior results section essentially refute this point.

Thank you for pointing out this inconsistency. The text has no been updated as follows on line 708:

“Neurons in the primate OFC represent the value of appetitive and aversive Pavlovian conditioned stimuli, suggesting a role for OFC in the subjective value signals that ultimately inform Pavlovian responding.”

14. Later in the discussion they go into detail as to why these two studies differed but omitted a major difference: Morrison and Salzman focused heavily on the period where monkeys were learning. By contrast, here the author threw out trials where monkeys were not showing discriminatory CRs. So, I have a couple of questions based on this: 1) how many trials on average did it take the monkeys to exhibit “learning” as assessed by CRs in the present study? On line 168 the author quotes 60-100 learning trials, but no more information on this is given. 2) Was there any relationship between the emergence of learning related CRs and gaze patterns? In that if learning occurred faster in a session was this associated with a higher correlation between CRs and eye movements? Alternatively, if the learning period is analyzed using a sliding window approach across trials does discriminatory looking behavior precede the onset of discriminatory CRs? 3) How do the main physiology results compare if the learning period is analyzed on its own (although I realize that there may not be enough statistical power to do this) or this period is included in the main analysis?

These are excellent questions. First, a quick note on Morrison and Salzman: In their 2009 paper, Morrison and Salzman show trial-by-trial correlation between OFC activity and CRs (their Figures 10C-F), but using data that exclude the learning phase. In fact, the only results in the 2009 paper involving learning are shown in Figure 10A-B. There is a separate publication by these authors from 2011 that does focus heavily on responses during learning (Neuron 71:6); but this paper does not address trial-by-trial correlation with CRs. In other words, by our reading of these papers, there is no explicit documentation of trial-by-trial correlations during the learning phase of the task.

That said, we agree that it is indeed possible that the relationship between spiking activity and CRs could depend on whether the cue-reward associations are stable, or are being learned. Unfortunately, the present study cannot address this question, because learning phase data were typically not collected. Learning trials were usually performed as the electrodes were being slowly lowered to the target depth each day, so there was no opportunity to observe OFC activity during learning. And CRs in learning trials were monitored on an ‘online’ basis only, with full data collection beginning only when the CRs were stable and commensurate with the cue values -- which typically took 60-100 trials, as described in the methods. All of this was by design: our goal was to only collect data in a stable behavioral regime, to minimize any ‘drift’ in the subjective value of the cues.

We wholeheartedly agree that the OFC could take on different roles during learning versus stable performance, but we think these questions would be best addressed in a study that is expressly designed around this question.

15. Are their CRs that the author thinks the OFC activity might be more related to? They briefly touch on autonomic responses but don't elaborate much more on this. They might want to note the other work showing that OFC lesions affect learning related autonomic responses and behavior (Reekie et al., PNAS, 2008).

Thank you for the suggestion, we have updated the discussion, beginning on line 794 as follows:

“More generally, lesion evidence implicates the OFC and nearby ventral and medial prefrontal areas in conditioned autonomic responses, including changes in heart rate (Reekie et al., 2008) and in pupil constriction in response to conditioned cues (Rudebeck et al., 2014; Hwang et al., 2018). This is consistent with anatomical evidence showing projections from these areas to lateral hypothalamic regions that innervate autonomic output centers in the brain and spinal cord (Barbas et al., 2003).”

16. In the discussion about how other areas outside of OFC could be involved in anticipatory licking, a motoric conditioned approach response, the author might want to have a look at the autoshaping literature in rodents. For instance, see Cardinal et al., Behavioral Neuroscience (2002). Another place to look could be the literature on volitional control of action/abulia which implicates the anterior cingulate cortex.

Thank you for the suggestion. The following has been added to the discussion, at line 845:

“Apart from the VS and vmPFC (which are specifically implicated in oromotor responses) other potential regions of interest include the amygdala and insular cortex, owing to their involvement in other forms of conditioned appetitive behavior such as autoshaping (Cardinal et al., 2002; Nasser et al., 2018), and the anterior cingulate cortex, owing to its role in motivation more generally (Cohen et al., 1999; Darby et al., 2018).”
